# Maspin Modulates Malignant Phenotypes Depending on Subcellular Localization in Pancreatic Ductal Adenocarcinoma Cell Lines

**DOI:** 10.3390/cancers18111815

**Published:** 2026-06-01

**Authors:** Hirotoshi Mochida, Tomohiko Sakabe, Takayuki Shingu, Karen Makishima, Yoshihisa Umekita

**Affiliations:** Department of Pathology, Faculty of Medicine, Tottori University, 86 Nishi-cho, Yonago 683-8503, Tottori, Japan; aeroheart@tottori-u.ac.jp (H.M.); sakabe@tottori-u.ac.jp (T.S.); takayuki412@tottori-u.ac.jp (T.S.); mkaren@tottori-u.ac.jp (K.M.)

**Keywords:** pancreatic ductal adenocarcinoma, mammary serine protease inhibitor, subcellular localization, ErbB signaling pathway

## Abstract

**Background/Objectives**: Mammary serine protease inhibitor (maspin) was originally identified as a tumor suppressor gene, as loss of its expression in breast cancer cell lines promotes invasiveness and tumorigenicity. In contrast, normal pancreatic ductal epithelium does not express the maspin protein, and its expression frequency increases as pancreatic intraepithelial neoplasia (PanIN) progresses to pancreatic ductal adenocarcinoma (PDAC). We have previously reported that cytoplasmic-only localization of maspin (cytMaspin) is an independent indicator of poor prognosis in patients with PDAC. However, the functional role of maspin in PDAC remains unclear. **Methods**: We investigated the subcellular localization and biological function of maspin in PDAC cell lines using Western blotting, immunofluorescence, RNA-seq, wound-healing assays, and invasion assays. **Results**: Endogenous maspin was detected in most PDAC cell lines, and RNA-seq was performed in S2-007 (panMaspin; nuclear and cytoplasmic localization of maspin) and S2-028 (cytMaspin), which were derived from the same parental cell line (SUIT-2). The invasive capability of S2-028 cells (cytMaspin) was higher than that of S2-007 cells (panMaspin), showing upregulation of the ErbB family and axon-guidance pathways. Furthermore, maspin overexpression in PANC-1 and S2-020 cells resulted in panMaspin and cytMaspin, respectively. PANC-1 cells overexpressing maspin showed decreased invasive capability via suppression of HER2 expression and Akt activation. **Conclusions**: Although maspin expression has generally been considered an unfavorable prognostic indicator in patients with PDAC, our findings suggest that its biological effects may differ depending on its subcellular localization. Specifically, nuclear localization is linked to less aggressive phenotypes, whereas cytoplasmic localization is associated with more malignant behavior.

## Simple Summary

Mammary serine protease inhibitor (maspin) is widely known as a tumor suppressor protein; however, we previously reported that cytoplasmic-only maspin (cytMaspin) expression in patients with pancreatic ductal adenocarcinoma (PDAC) is an independent unfavorable prognostic indicator. This study aims to elucidate the cellular function of maspin in PDAC, with a particular focus on its subcellular localization. We found that the PDAC cell line showing cytMaspin expression (S2-028) exhibited increased invasive capability and ErbB family signaling pathway activity compared with a cell line (S2-007) expressing nuclear and cytoplasmic maspin (panMaspin), both of which were derived from the same parent cell line (SUIT-2). We then established two PDAC cell lines stably overexpressing maspin (PANC-1, panMaspin, and S2-020, cytMaspin), showing decreased and increased migration capabilities, respectively, compared with control cell lines. These findings suggest that the biological effects of maspin in PDAC may differ depending on its subcellular localization.

## 1. Introduction

Pancreatic cancer has one of the poorest prognoses among malignancies worldwide, with a five-year relative survival rate of approximately 13% [1]. Epidemiological forecasts suggest that pancreatic cancer will rise to the second leading cause of cancer-related mortality in the United States by 2030 [2]. Pancreatic cancer is often diagnosed at an advanced stage, with low resectability and poor responsiveness to treatment, because early detection of lesions is difficult [3]. Therefore, elucidating the molecular mechanisms underlying pancreatic carcinogenesis and the relevant biological functions is imperative. Among pancreatic malignancies, pancreatic ductal adenocarcinoma (PDAC) accounts for the largest proportion based on the histopathological subtype [4]. PDAC and its precursor lesions, pancreatic intraepithelial neoplasia (PanIN), harbor several genetic alterations, including mutations in *HER2/neu*, *KRAS*, *p16*, *TP53*, *DPC4*, and *BRCA2* [5]. Despite active investigations into targeted therapies against these genes, the clinical results remain unsatisfactory, underscoring the need to identify novel molecular targets.

Mammary serine protease inhibitor (maspin) was originally identified as a tumor suppressor that inhibits tumor cell invasion, motility, and angiogenesis [6,7,8]. In several tissues, such as the breast [6] and prostate [9], normal epithelial cells express maspin protein, and its expression is often downregulated during carcinogenesis. In contrast, normal human pancreatic duct epithelial (HPDE) cells do not express maspin in the pancreas, whereas PDAC cells generally express maspin [10]. Higher maspin methylation was detected in normal HPDE cell lines than in normal mammary epithelial cell lines [11]. In addition, Maass et al. reported that almost all PDAC and PanIN grade 3 cases showed maspin expression, whereas maspin expression was absent in normal HPDE, ductal hyperplasia without dysplasia, and low-grade PanIN cases, suggesting that maspin expression differs from that in breast cancer, gastric cancer, and melanoma [12].

We previously reported, based on immunohistochemical studies of resected specimens of breast [13], lung [14], and pancreatic cancer [15], that patients whose tumors expressed maspin only in the cytoplasm (cytMaspin) had a poorer prognosis. Cao et al. reported that most PDAC cases showed maspin overexpression and that these patients had worse prognoses than cases lacking maspin expression, based on 223 surgically resected clinical specimens [10]. In addition, no significant difference in overall survival (OS) was detected between cytMaspin cases and cases with nuclear and cytoplasmic maspin expression (panMaspin). In contrast, we have reported that patients with cytMaspin expression showed a significantly shorter OS than patients with panMaspin in 92 surgically resected PDAC specimens [15]. In addition, nuclear localization of maspin has been reported to be essential for its tumor-suppressive function in certain human mammary carcinoma cell lines [16].

Thus, we hypothesized that panMaspin may be associated with tumor-suppressive function, whereas cytMaspin may be correlated with tumor aggressiveness in PDAC. The present study aimed to elucidate the functional role of maspin, which is dependent on its subcellular localization, in multiple pancreatic cancer cell lines.

## 2. Materials and Methods

### 2.1. Cell Culture, Reagents and Treatments

The immortalized human normal HPDE cell line H6c7 (HPDE6-E6E76c7) was purchased from Kerafast (Boston, MA, USA) and maintained in Keratinocyte SFM (Thermo Fisher Scientific, Waltham, MA, USA) containing 5 ng/mL EGF and 50 µg/mL bovine pituitary extract.

Human PDAC cell lines were obtained from the RIKEN BioResource Research Center (Ibaraki, Japan) (PANC-1), the Japanese Collection of Research Bioresources Cell Bank (Osaka, Japan) (MIA PaCa-2), the American Type Culture Collection (Manassas, VA, USA) (Capan-1, PSN-1, BxPC-3, AsPC-1, and HPAF-II), and Cell Resource Center for Biomedical Research, Institute of Development, Aging and Cancer, Tohoku University (Tohoku, Japan) (SUIT-2, S2-CP8, S2-VP10, S2-007, S2-013, S2-020, and S2-028), respectively.

PANC-1, PSN-1, BxPC-3, AsPC-1, and HPAF-II cells were maintained in Roswell Park Memorial Institute 1640 (RPMI1640; Thermo Fisher Scientific). Capan-1 cells were maintained in Iscove’s Modified Dulbecco’s Medium (IMDM; Thermo Fisher Scientific, Waltham, MA, USA). MIA PaCa-2, SUIT-2, S2-CP8, S2-VP10, S2-007, S2-013, S2-020, and S2-028 cells were maintained in Dulbecco’s modified Eagle’s medium (DMEM; Thermo Fisher Scientific, Waltham, MA, USA).

Only the medium for Capan-1 cells was supplemented with 20% heat-inactivated fetal bovine serum (FBS; Biological Industries, Cromwell, CT, USA), whereas all other media were supplemented with 10% FBS. All cell lines were maintained at 37 °C in a humidified incubator with 5% CO_2_ under standard culture conditions.

### 2.2. Real-Time Quantitative Polymerase Chain Reaction (RT-qPCR)

Total RNA was extracted from PDAC cell lines cultured in six-well plates using TRIzol Reagent (Thermo Fisher Scientific, Waltham, MA, USA). Complementary DNA (cDNA) was subsequently synthesized from the isolated RNA using the High-Capacity RNA-to-cDNA Kit (Thermo Fisher Scientific, Waltham, MA, USA). Quantitative real-time PCR analysis was performed on a LightCycler 96 System (Roche Diagnostics, Mannheim, Germany) using TaqMan Gene Expression Master Mix (Thermo Fisher Scientific, Waltham, MA, USA), following the manufacturer’s protocol. Details of the primer and probe sets employed in this study are provided in Supplementary Table S1. Relative mRNA expression levels were normalized to *ACTB*, which served as the endogenous control. Three independent experiments were conducted.

### 2.3. Transient Maspin Knockdown by Adenoviral shRNA

Transient knockdown of maspin was performed using adenoviral vectors expressing short hairpin RNAs (shRNAs) targeting human maspin or a non-targeting scrambled control. All vectors were custom-generated by VectorBuilder Inc. (Chicago, IL, USA). The scrambled control vector was sh-Scramble, and the two maspin-targeting vectors were shMaspin-1 and shMaspin-2. S2-007 cells were infected with adenoviral vectors at a multiplicity of infection of 250 under standard culture conditions for 24 h. After infection, the culture medium was replaced every 48 h.

### 2.4. Western Blotting

Cells were lysed in radioimmunoprecipitation assay buffer (Fujifilm Wako Pure Chemical Corporation, Osaka, Japan) supplemented with protease and phosphatase inhibitors (Merck Millipore, Billerica, MA, USA). For cell lines expressing endogenous maspin, proteins were fractionated into subcellular components using the Subcellular Protein Fractionation Kit for Cultured Cells (Thermo Fisher Scientific, Waltham, MA, USA) according to the manufacturer’s protocol. Protein concentrations were quantified using the XL-Bradford assay (APRO Science, Tokushima, Japan), and equivalent amounts of protein were loaded into each lane using a Hamilton syringe to minimize loading variability. Proteins were resolved by sodium dodecyl sulfate–polyacrylamide gel electrophoresis using 7.5–12.5% gels and subsequently transferred onto 0.45 µm polyvinylidene fluoride membranes. After transfer, membranes were cut based on molecular weight markers and probed separately with the indicated primary antibodies. After blocking for 90 min in 5% ECL Prime blocking agent (Cytiva, Tokyo, Japan), the membranes were incubated with the primary antibodies overnight at 4 °C. The primary antibodies used are listed in Supplementary Table S2.

Horseradish peroxidase-conjugated anti-mouse IgG or anti-rabbit IgG (both at a 1:30,000 dilution; Cell Signaling Technology, Danvers, MA, USA) were used. Primary antibodies against the ErbB family, phosphorylated Akt, and total Akt were diluted in Can Get Signal Immunoreaction Enhancer Solution 1, whereas the respective secondary antibodies were diluted in Solution 2 (TOYOBO, Osaka, Japan). All other antibodies were diluted in 5% ECL Prime blocking agent. After immunodetection, bound antibodies were removed using Stripping Solution (Fujifilm Wako Pure Chemical Corporation, Osaka, Japan), when required, to allow the same membrane to be reprobed with different antibodies. All assays were performed in at least three independent experiments. Signal intensities of total protein were normalized against β-actin, whereas phosphorylation levels were normalized against their corresponding total protein levels.

Immunoreactive signals were detected using the ECL Prime Western Blotting Detection Reagent (Cytiva, Tokyo, Japan), visualized with the Amersham ImageQuant 800 (Cytiva), and quantified using ImageJ/Fiji software (ver. 1.53k).

### 2.5. Establishment of PDAC Cell Lines Overexpressing Maspin

The procedures for generating lentiviral expression plasmids (pLenti/ZsGreen and pLenti/maspin-ZsGreen) and viral vectors have been described previously [17]. Lentiviral transduction was performed at a multiplicity of infection of 25 for PANC-1 and MIA PaCa-2 cells and 100 for PSN-1 and S2-020 cells. Stable integrants were selected by culturing cells in medium containing 10 µg/mL blasticidin (Fujifilm Wako Pure Chemical Corporation, Osaka, Japan). The following cell lines were established by pooling clones that survived in the presence of blasticidin: PANC-1-control (PANC-1-ctrl), PANC-1-maspin (PANC-1-masp), MIA PaCa-2-control (MIA PaCa-2-ctrl), MIA PaCa-2-maspin (MIA PaCa-2-masp), PSN-1-control (PSN-1-ctrl), PSN-1-maspin (PSN-1-masp), S2-020-control (S2-020-ctrl), and S2-020-maspin (S2-020-masp).

### 2.6. Immunofluorescence

All cell lines were cultured on Nunc Lab-Tek II 8-well chamber slides (Thermo Fisher Scientific, Waltham, MA, USA) and incubated overnight at 37 °C in a humidified incubator with 5% CO_2_. Samples were fixed with 4% paraformaldehyde in phosphate-buffered saline (PBS) for 15 min, followed by permeabilization with ice-cold 100% methanol at −20 °C for 10 min and subsequent treatment with 0.2% Triton-X in PBS for 5 min at room temperature (RT).

Non-specific binding was blocked by incubation with 3% bovine serum albumin in PBS at RT for 60 min. Samples were incubated with an anti-maspin primary antibody diluted in PBS at 4 °C overnight, followed by incubation with Alexa Fluor 488- (for endogenous maspin-positive cells) or Alexa Fluor 647 (for stable maspin-overexpression cells to avoid co-staining with ZsGreen)-conjugated secondary antibodies (Thermo Fisher Scientific, Waltham, MA, USA) at RT for 60 min. Nuclei were counterstained with 4′,6-diamidino-2-phenylindole at RT for 15 min. Autofluorescence was quenched using the Vector True View Autofluorescence Quenching Kit (Funakoshi Frontiers in Life Science, Tokyo, Japan), after which the samples were mounted with ProLong Diamond Antifade Mountant (Thermo Fisher Scientific, Waltham, MA, USA) to obtain slides. Fluorescence images were acquired using a Zeiss LSM780 confocal microscope (Carl Zeiss, Baden-Württemberg, Germany). Three independent experiments were conducted.

For evaluation of maspin subcellular localization, panMaspin- or cytMaspin-positive cells were counted at ×400 magnification in three randomly selected fields (*n* = 3), and the ratios were calculated.

### 2.7. Proliferation Assay

Cell Counting Kit-8 (DOJINDO Laboratories, Kumamoto, Japan) was used to assess the proliferation of PDAC cell lines according to the manufacturer’s protocol. For each cell line, the following number of cells were seeded into 96-well plates: PSN-1, 5.0 × 10^3^ cells/well; MIA PaCa-2 and S2-020, 7.5 × 10^3^ cells/well; and PANC-1, 1.0 × 10^4^ cells/well. After culturing in standard culture medium, 10 μL of CCK-8 reagent was added per 100 μL culture medium.

Cells were further incubated at 37 °C in a humidified atmosphere with 5% CO_2_ for 30 min (MIA PaCa-2), 50 min (S2-020), and 60 min (PANC-1 and PSN-1). Cell proliferation was assessed by quantifying optical density at 450 nm using an Infinite M Nano microplate reader (TECAN, Männedorf, Switzerland). Measurements were taken at 6, 24, 48, and 72 h post-seeding. Three independent experiments were conducted.

### 2.8. Cell Invasion Assay

Following 24 h of serum deprivation, PDAC cell lines were introduced into the upper compartments of transwell inserts (8 µm pore size) placed in 24-well plates, with serum-free medium maintained in the upper chambers. The lower compartments were filled with IMDM supplemented with 20% heat-inactivated FBS to establish a chemoattractant gradient.

Cells were allowed to invade for 48 h under standard culture conditions. At the end of the incubation period, cells remaining on the upper surface of the membranes were carefully removed with cotton swabs. The membranes were subsequently fixed in 4% paraformaldehyde for 15 min, and cells that had traversed the membrane were visualized by crystal violet staining using the QCM ECMatrix Cell Invasion Assay kit (24-well, 8 µm; Merck Millipore, Billerica, MA, USA) according to the manufacturer’s protocols.

Entire membrane surfaces were imaged using a BZ-X800 all-in-one fluorescence microscope (KEYENCE, Osaka, Japan), and invaded cells were quantified using ImageJ/Fiji software (version 1.53k). Three independent experiments were conducted.

### 2.9. Cell Migration Assay

Stable PDAC cell lines were exposed to mitomycin C (10 µg/mL) for 2 h to inhibit cell proliferation. The treated cells were then placed into culture inserts of the ibidi Culture-Insert 2 Well in µ-Dish^35mm^, high ibiTreat (ibidi GmbH, Gräfelfing, Germany) and maintained overnight at 37 °C in a humidified atmosphere containing 5% CO_2_.

Following cell attachment, the culture inserts were gently removed using flame-sterilized tweezers, and the dishes were replenished with complete culture medium and further incubated. Images were obtained using an IX73 inverted microscope (Olympus Corporation, Tokyo, Japan) at 0 and 12 h (PANC-1-ctrl, PANC-1-masp, PSN-1-ctrl, and PSN-1-masp), and 36 h (MIA PaCa-2-ctrl, MIA PaCa-2-masp, S2-020-ctrl, and S2-020-masp) after incubation.

The wound area was measured using the *Wound_healing_size_tool* plugin [18] in ImageJ/Fiji software. Wound closure was determined by normalizing the wound area at each time point to the initial gap area at 0 h and calculated using the following equation: Wound closure (%) = [(wound area at 0 h − wound area at time x)/wound area at 0 h] × 100. For all experiments, at least three independent experiments were conducted.

### 2.10. Transcriptome Analysis (RNA-Seq)

Total RNA was extracted from S2-007 and S2-028 cells using the RNeasy Mini Kit (QIAGEN, Valencia, CA, USA) according to the manufacturer’s protocols. Purified RNA samples were submitted to Eurofins Genomics (Tokyo, Japan) for RNA-Seq analysis. Following poly (A) enrichment, RNA was fragmented and reverse-transcribed into cDNA using random primers. Strand-specific mRNA libraries were constructed using adapter ligation and fragmentation. RNA-Seq was performed on a NovaSeq 6000 platform (Illumina, San Diego, CA, USA).

Raw sequencing reads were quality-filtered using Trimmomatic software (ver. 0.39) and aligned to the human reference genome (GRCh38.p14) using BWA (ver. 0.7.17). Gene-level read counts were normalized using the trimmed mean of M-values method, a process performed with the aid of EdgeR software (ver.4.3.1). Following this, statistical analysis was conducted using a likelihood ratio test, and genes with a false discovery rate-adjusted *p* < 0.05 were considered statistically significant. In the comparison of S2-028 with S2-007, genes with log2 (fold change) ≥ 0.585 or ≤−0.585 were defined as upregulated and downregulated genes, respectively. The RNA-seq datasets generated by Eurofins have been deposited in the NCBI Gene Expression Omnibus under accession number GSE314358. To gain insight into the biological functions of the differentially expressed genes (DEGs), gene ontology (GO) and biological process (BP) analyses, as well as Kyoto Encyclopedia of Genes and Genomes (KEGG) pathway enrichment analyses, were conducted using the Database for Annotation, Visualization, and Integrated Discovery (DAVID, https://davidbioinformatics.nih.gov/, accessed on 29 April 2026). To this end, DEGs were subjected to a series of analyses, including tree mapping, to elucidate their biological functions. This analysis was facilitated by the online Reduce & Visualize Gene Ontology (REViGO, http://revigo.irb.hr/, accessed on 29 April 2026) tool.

### 2.11. Gene Set Enrichment Analysis

Gene set enrichment analysis (GSEA) was performed using GSEA software (version 4.4.0 for Windows) obtained from the Molecular Signatures Database (MSigDB). RNA sequencing (RNA-seq) data derived from S2-007 and S2-028 cell lines (*n* = 3 per group) were analyzed to identify pathways differentially enriched between the two conditions.

Gene sets were obtained from the KEGG Medicus collection (c2.cp.kegg_medicus.v2026.1.Hs.symbols.gmt) available in MSigDB. Genes were ranked according to the signal-to-noise ratio metric implemented in the GSEA algorithm. Enrichment scores were calculated for each gene set, and statistical significance was assessed using phenotype-based permutation testing with 1000 permutations. The enrichment scores were normalized to generate normalized enrichment scores (NES), and multiple hypothesis testing was controlled using the false discovery rate (FDR). Gene sets with an FDR q-value < 0.25 were considered significantly enriched. The detailed GSEA results are provided in Supplementary Table S3.

### 2.12. Statistical Analysis

Data are presented as the mean ± standard deviation. Statistical differences between group means were assessed using Student’s *t*-test and Dunnett’s test. Statistical significance was set at *p* < 0.05.

## 3. Results

### 3.1. The Expression Status and Subcellular Localization of Maspin in PDAC Cell Lines

**Figure 1:**
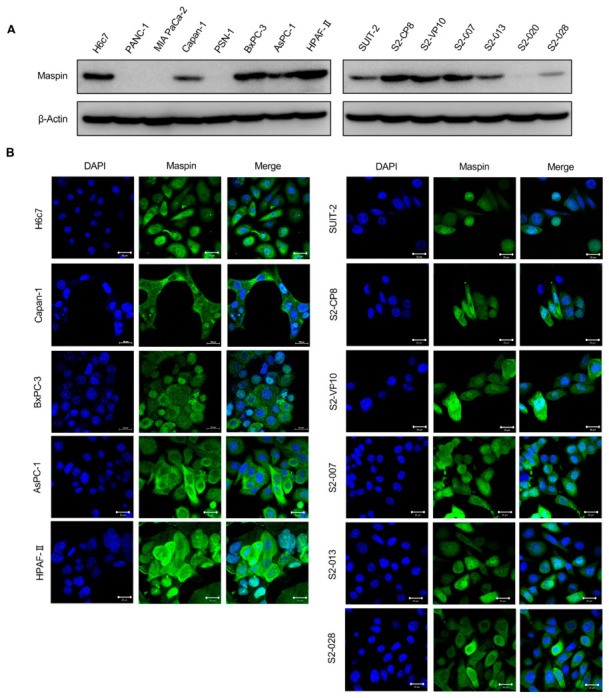

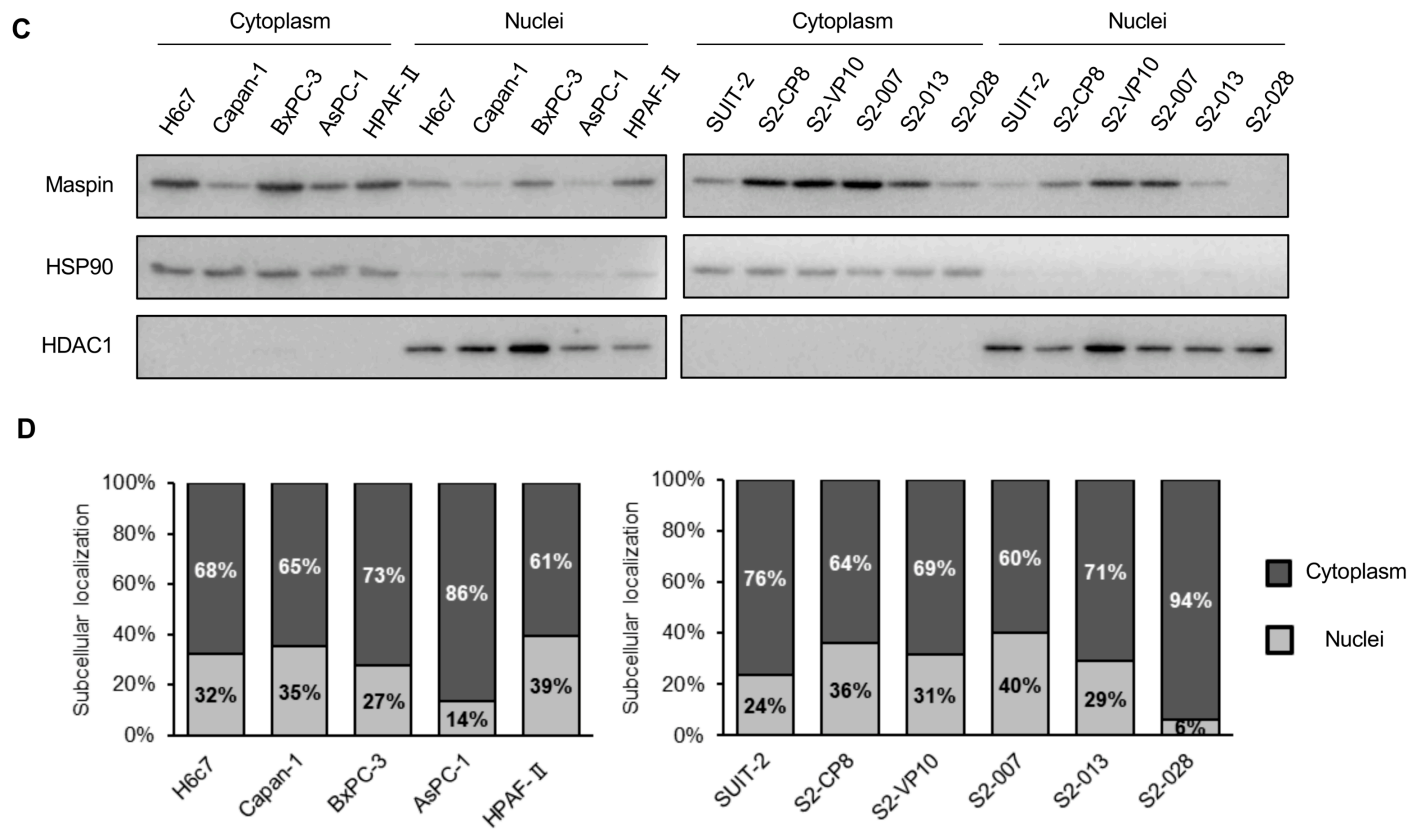
Maspin expression in human PDAC cell lines and H6c7 (an immortalized human normal pancreatic duct epithelial cell line). (**A**) Maspin and β-actin (loading control) protein levels in whole-cell lysates from 14 PDAC cell lines and the H6c7 cell line. Whole western blot images are shown in Supplementary Figure S1. (**B**) Representative images showing subcellular localization of maspin by immunofluorescence staining in maspin-positive cell lines. Scale bar, 20 μm. (**C**) Expression levels of maspin in the cytoplasmic and nuclear fractions. HSP90 and HDAC1 were used as loading controls for the cytoplasmic and nuclear fractions, respectively. Whole Western blot images are shown in Supplementary Figure S1. (**D**) Percentage of maspin localization in the cytoplasm and nuclei for each cell line. The graph represents averages from Western blot analysis. Data are presented as the mean from three independent experiments.

To determine the expression levels of maspin in PDAC cell lines, whole-cell lysates were analyzed using Western blotting. Among the 15 cell lines, including the normal HPDE cell line H6c7, maspin protein expression was suppressed in PANC-1, MIA PaCa-2, and PSN-1, and was only weakly detected in S2-020. Conversely, maspin expression was observed in the remaining 11 cell lines, including H6c7 cells (Figure 1A).

Immunofluorescence (IF) staining revealed that maspin was predominantly expressed in the cytoplasm (cytMaspin) in AsPC-1 and S2-028, whereas it was detected in both the nucleus and cytoplasm (panMaspin) in H6c7 and the other PDAC cell lines (Figure 1B). Furthermore, the results of the Western blot analysis of protein samples fractionated into nuclear and cytoplasmic components were consistent with those obtained from IF staining (Figure 1C,D). Notably, none of the cell lines exhibited nuclear predominant subcellular localization of maspin.

These results suggest that the subcellular localization of maspin in PDAC cell lines is predominantly panMaspin in most cell lines, whereas some cell lines exhibit suppressed expression or a shift toward cytoplasmic-predominant localization.

### 3.2. Differences in Cell Proliferative and Invasive Capability Between S2-007 and S2-028 Cell Lines

**Figure 2:**
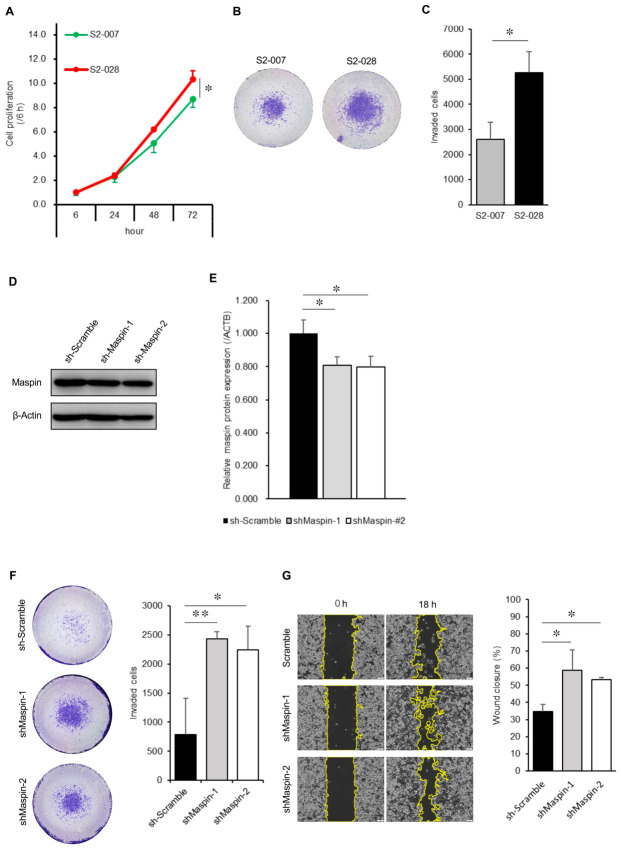
Characterization of S2-007 (panMaspin) and S2-028 cells (cytMaspin) and functional analyses following transient maspin knockdown. (**A**) Cell proliferation of S2-007 and S2-028 cell lines was measured at 6 h, 24 h, 48 h, and 72 h; the data were normalized to the 6 h value. Data are shown as the mean ± SD (*n* = 3). * *p* < 0.05, S2-007 vs. S2-028; Student’s *t*-test. (**B**) S2-007 and S2-028 cells were seeded into transwells and, after 48 h of incubation, invasion was evaluated by a colorimetric method. Representative images of wells from the transwell invasion assay for S2-007 and S2-028 cells (*n* = 3). (**C**) Invaded cell counting was performed using ImageJ. Data are shown as the mean ± SD (*n* = 3). * *p* < 0.05, S2-007 vs. S2-028; Student’s *t*-test. (**D**) Western blot analysis of maspin protein expression in S2-007 cells following transient maspin knockdown. ACTB was used as a loading control (*n* = 3). Whole Western blot images are shown in Supplementary Figure S2. (**E**) Quantification of maspin protein expression normalized to ACTB in S2-007 cells following transient maspin knockdown. Data are presented as mean ± SD (*n* = 3). * *p* < 0.05. (**F**) Representative images of invasion assays in S2-007 cells following transient maspin knockdown (*n* = 3). Quantification of invaded cells was performed using ImageJ. * *p* < 0.05, ** *p* < 0.01. (**G**) Representative images of wound-healing assays in S2-007 cells transduced with sh-Scramble, shMaspin-1, or shMaspin-2. Images were obtained at 0 h and 18 h after scratch induction. Yellow lines indicate wound margins, and the bar graph represents the percentage of wound closure after 18 h. Quantification was performed using ImageJ. Data are shown as the mean ± SD (*n* = 3). * *p* < 0.05, S2-007 vs. S2-028; Student’s *t*-test. Scale bar, 100 μm.

S2-007 and S2-028 cell lines are highly invasive sublines derived from the parental SUIT-2 cell line [19,20]. To investigate the biological differences between the two cell lines exhibiting different subcellular localizations of maspin, we compared their proliferative and invasive capabilities. As shown in Figure 2A, the proliferative capability of S2-028 cells was significantly higher than that of S2-007 cells (*p* < 0.05). Furthermore, the invasive capability of S2-028 cells was significantly greater than that of S2-007 cells, consistent with their proliferative capability (*p* < 0.05) (Figure 2B,C).

### 3.3. Functional Effects of Transient Maspin Knockdown in S2-007 Cells

To complement the gain-of-function analyses, transient loss-of-function experiments were performed using adenoviral shRNA vectors targeting maspin in endogenous maspin-positive PDAC cell lines. Quantitative real-time PCR demonstrated a significant reduction in maspin mRNA expression in both S2-007 cells transduced with two independent shRNAs (shMaspin-1 and shMaspin-2), compared with the scramble control (Supplementary Figure S2). Consistent with these findings, Western blot analysis confirmed a decreased maspin protein expression in both cell lines following shRNA-mediated knockdown (Figure 2D,E). In S2-007 cells, invasion assays showed significantly increased invasive capability following maspin suppression (Figure 2F). In addition, wound-healing assays revealed significantly enhanced migratory activity after maspin knockdown compared with sh-Scrambled controls (Figure 2G).

These findings suggest that endogenous panMaspin may contribute to the suppression of invasive and migratory phenotypes in the S2-007 cell line.

### 3.4. Comparison of Gene Expression Profiles in PDAC Cell Lines with Different Subcellular Localizations of Maspin

**Figure 3:**
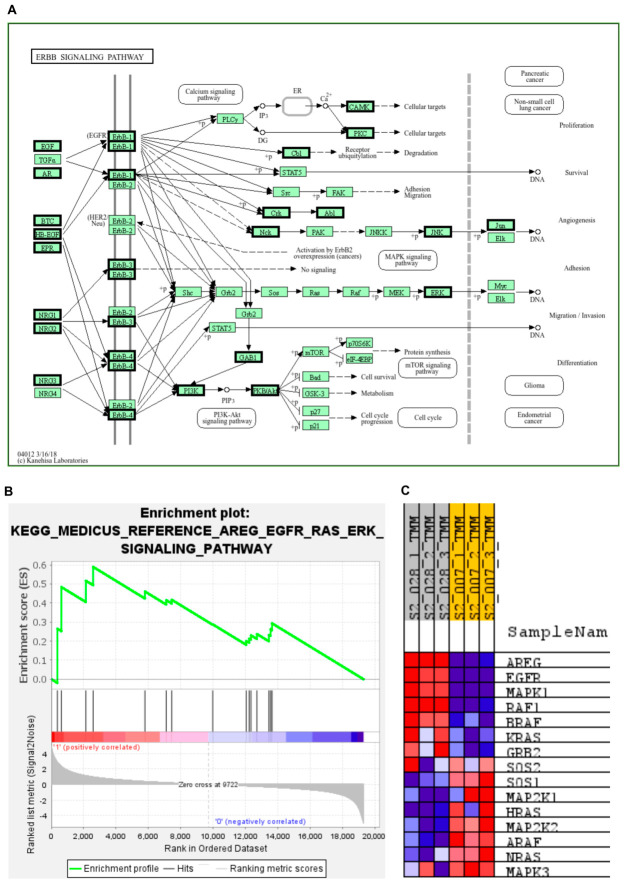

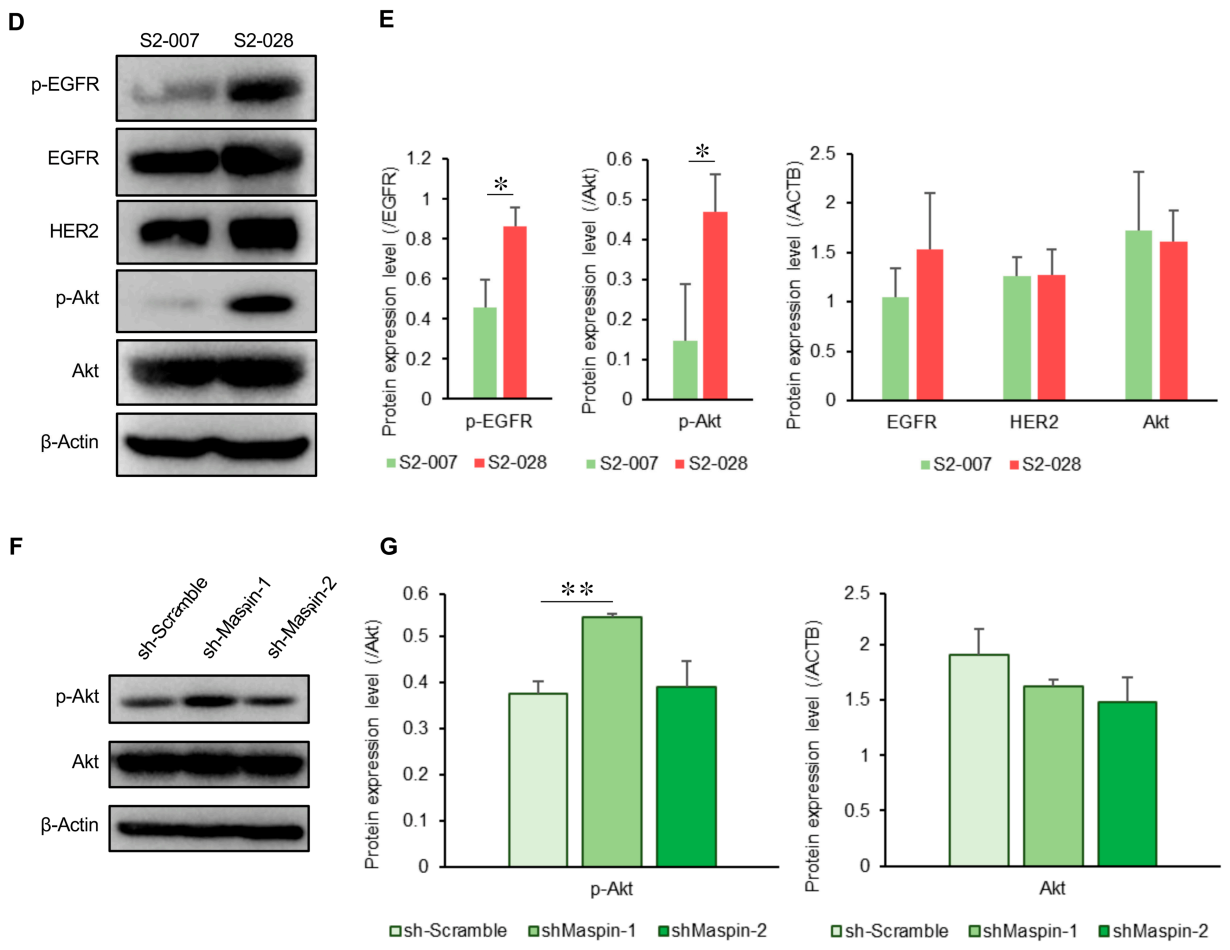
Distinct malignant phenotypes and ErbB-related signaling profiles in S2-007 (panMaspin) and S2-028 (cytMaspin) cells. (**A**) ErbB signaling pathway (KEGG Pathway) in S2-028 cells compared to S2-007 cells. Thick black borders represent genes that are significantly upregulated in S2-028 cells compared with S2-007 cells. (**B**) Gene set enrichment analysis (GSEA) plot showing significant enrichment of the KEGG_MEDICUS_REFERENCE_AREG_EGFR_RAS_ERK_SIGNALING_PATHWAY gene set in S2-028 cells relative to S2-007 cells. (**C**) Heatmap of representative genes involved in the AREG–EGFR–RAS–ERK signaling pathway in S2-028 and S2-007 cells based on RNA-seq data. Red and blue indicate higher and lower expressions, respectively. (**D**) Expression and phosphorylation levels of ErbB family members and Akt in S2-007 (panMaspin) and S2-028 cells (cytMaspin). Whole Western blot images are shown in Supplementary Figure S4. (**E**) Quantification of ErbB family and Akt protein levels in S2-007 (panMaspin) vs. S2-028 cells (cytMaspin). Bar graph generated by image quantification in ImageJ. Data are shown as the mean ± SD (*n* = 3). * *p* < 0.05, S2-007 vs. S2-028; Student’s *t*-test. (**F**) Expression and phosphorylation levels of ErbB family members and Akt in S2-007 cells following maspin knockdown. Whole Western blot images are shown in Supplementary Figure S5. (**G**) Quantitative analysis of expression and phosphorylation levels of EGFR, HER2, and Akt in S2-007 cells following maspin knockdown. Protein levels were quantified using ImageJ/Fiji and normalized to β-actin or corresponding total protein levels. Data are presented as mean ± SD from three independent experiments (*n* = 3). ** *p* < 0.01; Dunnett’s test.

Transcriptome analysis was conducted on S2-007 (panMaspin) and S2-028 cells (cytMaspin), sublines established from the same parent SUIT-2 cell line [19], to investigate the gene expression profiles in cell lines exhibiting different subcellular localizations of maspin. A total of 5012 genes were identified as significant DEGs between S2-007 and S2-028 cells. Of these genes, 2778 and 2234 genes were significantly upregulated and downregulated, respectively (Supplementary Table S3, Supplementary Figure S3A). Gene ontology analysis of these DEGs revealed that the upregulated genes in the S2-028 cells were enriched in 10 GO-BP terms, whereas downregulated genes were enriched in 12 GO-BP terms. The REViGO tool was used to summarize and visualize GO terms. GO-BP terms that were upregulated in S2-028 cells were categorized into four distinct clusters using TreeMap (Supplementary Figure S3B). Several GO terms were associated with axon guidance and cell migration. Downregulated GO-BP terms were categorized into six distinct clusters, including positive regulation of T cell activation (Supplementary Figure S3C). KEGG pathway enrichment analysis revealed that the expression levels of genes involved in the ErbB signaling pathway and Pancreatic cancer pathway were higher in S2-028 cells compared with S2-007 cells (Figure 3A, Supplementary Figure S3D, Supplementary Table S4). Therefore, phosphorylation profiles of these proteins were examined in S2-007 and S2-028 cells by Western blotting. Gene set enrichment analysis (GSEA) revealed that the KEGG_MEDICUS_REFERENCE_AREG_EGFR_RAS_ERK_SIGNALING_PATHWAY gene set was significantly enriched in S2-028 cells compared with S2-007 cells (NES = 1.70). The enrichment plot demonstrated a positive enrichment score, indicating that genes associated with AREG–EGFR–RAS–ERK signaling were preferentially clustered at the top of the ranked gene list (Figure 3B). Consistent with the GSEA results, heatmap visualization highlighted distinct expression patterns of genes involved in the AREG–EGFR–RAS–ERK signaling pathway between S2-028 and S2-007 cells (Figure 3C, Supplementary Table S5).

S2-028 cells demonstrated significantly higher phosphorylation of EGFR and Akt than S2-007 cells. (Figure 3D,E). To further investigate the relationship between endogenous maspin expression and ErbB-related signaling, quantitative analysis showed that the phosphorylation of Akt was significantly increased in shMaspin-1-treated cells, whereas total Akt expression showed no significant difference among groups (Figure 3F,G).

### 3.5. Establishment of Maspin-Reexpressing PDAC Cell Lines and Evaluation of Their Subcellular Localization

**Figure 4:**
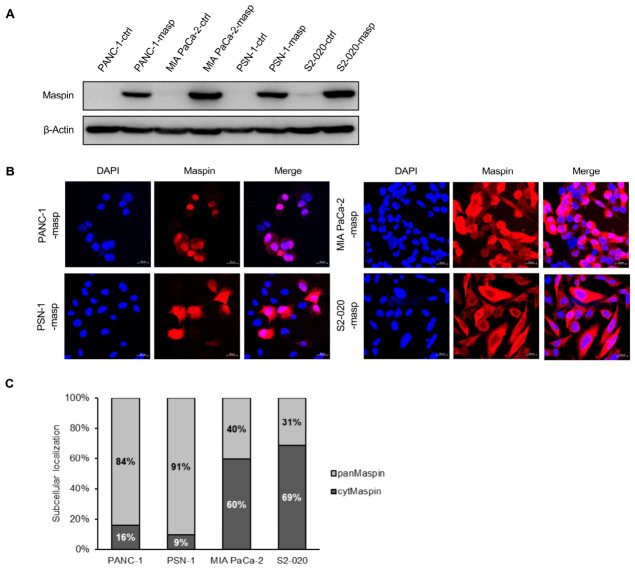
Establishment of PDAC cell lines overexpressing maspin exhibiting different intracellular localization. (**A**) Maspin and β-actin (loading control) protein levels in whole-cell lysates from four PDAC cell lines stably transfected with ZsGreen (-ctrl) or maspin-ZsGreen (-masp). Whole Western blot images are shown in Supplementary Figure S6. (**B**) Representative images showing subcellular localization of maspin by immunofluorescence staining in representative panMaspin cells (stably expressing: PANC-1, PSN-1) and cytMaspin cells (stably expressing: MIA PaCa-2, S2-020). Scale bar, 20 μm. (**C**) Ratio of cytMaspin to panMaspin in immunofluorescence-stained samples. Data are presented as the mean from three independent experiments.

To investigate the effects of increased maspin expression, stable maspin-expressing PDAC cell lines were established from PDAC cell lines with low endogenous maspin expression, including PANC-1, MIA PaCa-2, PSN-1, and S2-020 (designated PANC-1-masp, MIA PaCa-2-masp, PSN-1-masp, and S2-020-masp). The established cell lines showed markedly higher maspin expression levels than their respective control cell lines (Figure 4A). These cell lines displayed two distinct maspin subcellular localization patterns: PANC-1-masp and PSN-1-masp were classified as panMaspin, whereas MIA PaCa-2-masp and S2-020-masp were classified as cytMaspin (Figure 4B,C).

### 3.6. Functional Analysis of Established panMaspin and cytMaspin Cell Lines

**Figure 5:**
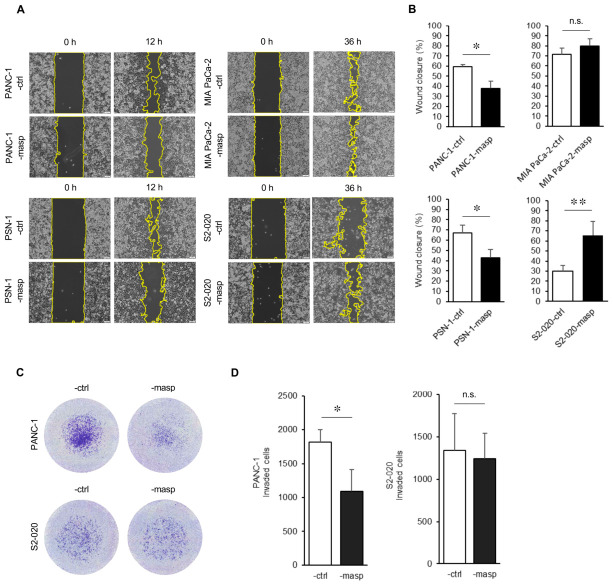

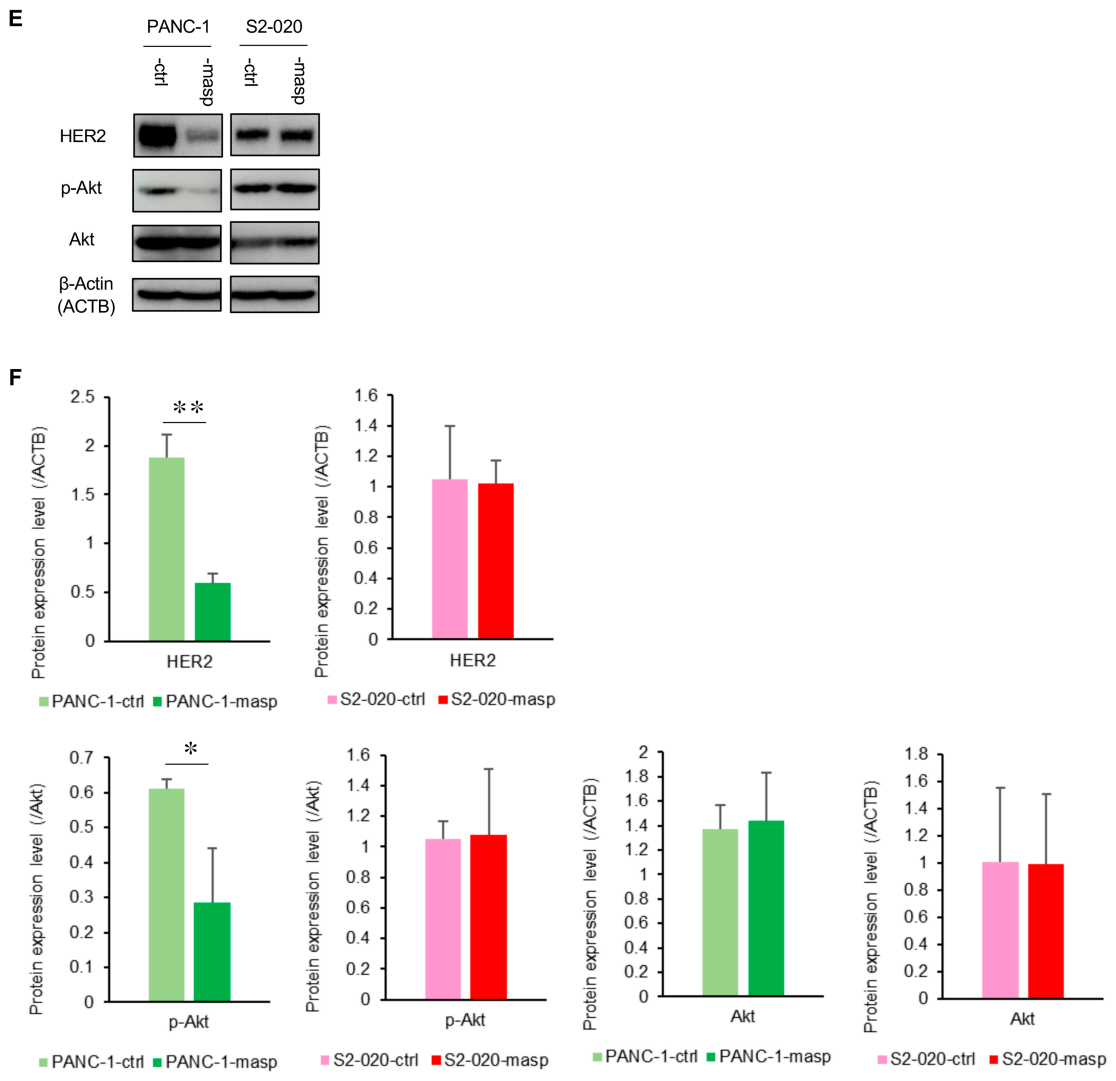
Characterization of established PDAC cell lines. (**A**) Representative images of wound-healing assays performed using PANC-1, PSN-1, MIA PaCa-2, and S2-020 cells stably transfected with ZsGreen (-ctrl) or maspin-ZsGreen (-masp) are shown. Scale bar, 100 μm. (**B**) The bar graph represents the percentage of wound closure after 12 and 36 h for PANC-1, PSN-1, MIA PaCa-2, and S2-020 cells stably transfected with ZsGreen (-ctrl) or maspin-ZsGreen (-masp), respectively. * *p* < 0.05, ** *p* < 0.01, vs. ctrl; Student’s *t*-test (*n* = 3). n.s., no significance. (**C**) Transfectant (PANC-1 and S2-020) cells were seeded into a transwell, and after 48 h of incubation, invasion was evaluated using a colorimetric method. Representative well images of a transwell invasion assay of PANC-1 and S2-020 cells (*n* = 3). (**D**) Invaded cell count was performed using ImageJ. Data are shown as the mean ± SD (*n* = 3). * *p* < 0.05, control vs. maspin-ZsGreen; Student’s *t*-test. (**E**) Expression and phosphorylation levels of ErbB family and Akt in transfectants of PANC-1 and S2-020 cells. Whole Western blot images are shown in Supplementary Figure S7. (**F**) Quantification of ErbB family and Akt protein levels in established stable PANC-1 and S2-020 cells using Western blotting. Bar graph shows image quantification in ImageJ. Data are shown as the mean ± SD (*n* = 3). * *p* < 0.05, ** *p* < 0.01; Student’s *t*-test.

No significant differences in cell proliferative capability were detected among any of the established cell lines when compared with their respective controls, regardless of the subcellular localization of maspin (Supplementary Figure S7). Additionally, to investigate the migratory capability of these cell lines, a wound-healing assay was conducted (Figure 5A,B). Migratory capability was significantly decreased in both PANC-1-masp and PSN-1-masp cells, which express panMaspin, compared with their respective control cells. Conversely, S2-020-masp cells expressing cytMaspin exhibited enhanced migratory capability compared to the control, whereas no significant difference was observed in MIA PaCa-2-masp cells.

To investigate the impact of panMaspin and cytMaspin on cell invasion, PANC-1-masp and S2-020-masp cells were selected as representatives of panMaspin and cytMaspin expression, respectively, and an invasion assay was performed. Compared with control cells, the number of invading cells was significantly reduced in PANC-1-masp cells, but not in S2-020-masp cells (Figure 5C,D). Furthermore, the phosphorylation levels of proteins involved in the EGFR signaling pathway in these cell lines were analyzed. Comparison within each cell line revealed that PANC-1-masp (panMaspin model) cells exhibited decreased HER2 protein and phosphorylated Akt levels relative to PANC-1-ctrl cells, whereas S2-020-masp cells (cytMaspin model) showed no change (Figure 5E,F). Except for differences in HER2 levels, the protein expression and phosphorylation profiles were not significantly altered by panMaspin or cytMaspin overexpression in either cell line. These results suggested that suppression of the ErbB family and its downstream Akt signaling is involved in the antitumor activity of panMaspin.

## 4. Discussion

PDAC is an aggressive cancer with a poor prognosis, and genetic mutations have been implicated in its carcinogenesis. The most frequently observed genetic alterations include mutations in the KRAS gene (85–95% of cases) [21] and inactivation of the tumor suppressor gene SMAD4, which occurs in approximately 55% of cases [22]. SMAD4, located on chromosome 18 (18q21.1), and its loss [12,23,24] or mutations [25,26] are involved in PDAC carcinogenesis. Similarly, maspin (SERPINB5), located on chromosome 18 (18q21.3), was initially identified as a tumor suppressor gene due to its expression in normal human mammary cell lines and tissues, and its absence in human breast cancer cell lines and tissues [6]. In contrast, maspin is not expressed in normal HPDE cells, whereas its expression increases during progression from PanIN to PDAC [10]. In our study, H6c7 and ten types of PDAC cell lines showed maspin protein expression, whereas expression was weak or suppressed in four PDAC cell lines. Fitzgerald et al. demonstrated that normal pancreatic tissue, as well as PANC-1 and MIA PaCa-2 cells, exhibit heavy methylation and hypoacetylation in the maspin promoter region, whereas treatment with 5-Aza-dC induced high maspin expression. Conversely, maspin-positive pancreatic cancer cell lines show demethylation and hyperacetylation of histone H3/H4 in the maspin promoter region, suggesting that methylation is the underlying cause of the epigenetic regulation of maspin expression [11]. These studies support our observation, and the loss of maspin expression in PSN-1 and S2-020 cells is likely due to methylation of the promoter region. Furthermore, maspin negativity can be caused by factors other than methylation, including increased epithelial–mesenchymal transition (EMT). Tang et al. demonstrated that maspin suppressed EMT by inhibiting endogenous HDAC1, regulating chromatin accessibility to transcription factors, and altering the cytoskeleton. In various cell lines, including those from gastric cancer, maspin increases E-cadherin expression and decreases vimentin expression, thereby inhibiting EMT [27]. Furthermore, it has been reported that Snail directly acts on the maspin promoter to suppress maspin expression, thereby promoting migration and invasion [27]. Additionally, the chromatin remodeling complex CBP/p300 reduces the binding of Ets-1 and c-Jun to the maspin promoter, causing maspin deficiency and consequently inducing a malignant phenotype [27]. The normal HPDE cell line H6c7 (HPDE/E6E7), which was established by immortalization via the induced expression of the HPV16-E6E7 gene, inhibiting p53/Rb, exhibited high levels of maspin and panMaspin expression [28]. Shachar et al. demonstrated that the concurrent administration of doxorubicin and cisplatin chemotherapy and E2F1 activation (ER-E2F1) in two osteosarcoma cell lines with different p53 phenotypes resulted in maspin overexpression and apoptosis induction [29]. These reports support our data and suggest that the induction of maspin expression in the H6c7 cell line is associated with the inactivation of Rb by HPV16-E7 and the activation of E2F1.

We previously reported that increased cytoplasmic maspin expression promotes EMT in breast cancer cell lines [17], and that lung [30] and pancreatic [15] cancer cell lines with cytMaspin tend to exhibit higher invasive capability. To our knowledge, this is the first study to compare two models derived from SUIT-2—the panMaspin model (S2-007) and the cytMaspin model (S2-028)—that differ in the subcellular localization of endogenous maspin protein. Notably, the cytMaspin model demonstrated significantly higher invasive capability. In the cytMaspin model, a significantly increased expression of EGFR, along with downstream Akt, was observed. In addition, phosphorylation of Akt (S473) was detected, indicating the activation of these pathways. Genes associated with GO terms related to cancer proliferation and metastasis (e.g., cell migration) were upregulated. Conversely, downregulated genes were significantly enriched in GO terms associated with tumor-suppressive processes such as positive regulation of T cell activation. These findings suggest that changes in the expression of these gene clusters promote the malignant properties of S2-028, including its invasive capability. Pathway analysis of the cytMaspin model (S2-028) revealed increased expression of the ErbB family and its downstream Akt in the ErbB signaling pathway. This suggests that ErbB signaling may further enhance malignant properties. The present GSEA results provide additional support for our hypothesis that cytoplasmic maspin localization is associated with a more aggressive molecular phenotype. In particular, the enrichment of the AREG–EGFR–RAS–ERK pathway in S2-028 cells supports a transcriptional state associated with enhanced receptor tyrosine kinase signaling and malignant phenotypes. However, since S2-007 and S2-028 may harbor distinct cell line-specific regulatory mechanisms, the observed pathway activation cannot be attributed exclusively to maspin subcellular localization. Although the mechanistic relationship between maspin expression and ErbB signaling is likely more complex, given that EGFR signaling can also activate the PI3K–Akt pathway, these findings remain consistent with the Akt-related signaling alterations observed in our protein analyses. Additionally, increased expression of the axon-guidance pathway was observed. Biankin et al. previously demonstrated the importance of this pathway in a large-scale pancreatic cancer genome study involving 142 patients with resectable PDAC (stages I and II) [31]. In PDAC, SLIT/ROBO signaling is considered crucial for regulating MET and WNT signaling, and loss of ROBO1/2 leads to the nuclear translocation of β-catenin, resulting in reduced complex formation with E-cadherin and decreased cell adhesion, which in turn enhances WNT signaling [31]. We found that *ROBO2* was significantly reduced in the cytMaspin model compared to the panMaspin model (Supplementary Table S3). These findings support our present and previous results that the cytMaspin model exhibits a higher invasive capability than the panMaspin model and that cytoplasmic localization of maspin in PDAC specimens is an independent poor prognostic factor for patients [15].

In contrast, maspin was overexpressed using lentivirus in maspin-negative cell lines. Cells with re-expressing panMaspin showed suppressed migration and cell invasion. This suggests that panMaspin has tumor-suppressive functions. Furthermore, cells expressing panMaspin showed reduced HER2 expression. Larbouret et al. reported that the inhibition of EGFR/HER2 heterodimers using antibody therapy produced a clear antitumor effect in a mouse model [32]. Furthermore, HER2 protein expression levels in PDAC patients have been reported to be equivalent to or higher than those in HER2-low breast cancer in more than half of the cases [33]. HER2 expression is also a poor prognostic factor in PDAC patients [34]. Collectively, these findings and these reports suggest that maspin, which is primarily expressed in the nucleus, may suppress the invasive capability by reducing the expression of HER2.

Although cytMaspin re-expression enhanced migration in S2-020 cells, no significant changes were observed in invasive capability or ErbB-related signaling markers. This suggests that cytoplasmic maspin expression alone may not be sufficient to reproduce the full phenotype of cells with endogenous cytoplasmic maspin. Differences between endogenous and re-expression models may reflect the broader cellular context and additional molecular alterations. Further studies using additional cytMaspin-expressing cell lines will be required to clarify the relationship between cytMaspin, ErbB signaling, and invasive behavior. In addition, the variability in migration assay results was observed between MIA PaCa-2 and S2-020 cells, despite both being classified as cytoplasmic maspin-expressing lines. This suggests that factors beyond maspin subcellular localization, such as intrinsic differences in genetic background or signaling networks, may influence migratory behavior. Therefore, maspin localization should be considered as one contributing factor within a more complex regulatory framework. In this study, invasion assays and phosphorylation analyses were not performed for the other two maspin-overexpressing cell lines, namely PSN-1 and MIA PaCa-2. Therefore, the extent to which our findings can be generalized across different cellular contexts remains to be established. Further studies using a broader range of cell lines will be necessary to confirm the consistency of these observations. In the present study, the functional differences observed among the cell lines were not entirely uniform, suggesting that factors beyond the subcellular localization of maspin may contribute to this phenotypic variability. Specifically, biological characteristics of SUIT-2-derived cell lines may influence their migratory and invasive capabilities. Therefore, the localization of maspin should be viewed as one contributing factor within a broader regulatory context, and further studies are required to clarify its relative contribution. In cell lines with endogenous maspin expression, the cytMaspin model S2-028 exhibited higher EGFR signaling activity than the panMaspin model S2-007. Longhi et al., using normal human mammary cell lines, demonstrated that EGF induces maspin nuclear localization [35] and reported that the key pathways involved in the nuclear translocation of maspin are EGFR, PI3K-Akt, and JAK2/STAT3 [36]. The expression levels of phosphorylated Akt were significantly reduced by maspin overexpression in PANC-1 cells, whereas no significant change was observed in S2-020 cells. S2-020 cells possess high levels of phosphorylated Akt, as do S2-028 cells, which is another endogenous cytMaspin model. These findings suggest that alterations in the expression profiles and rewiring of signaling pathways involving molecules such as the ErbB family and Akt, which occur during carcinogenesis, may have modified the subcellular localization and function of maspin. Conversely, this study revealed variations in phenotypic outcomes among experimental models, likely reflecting cell line-specific molecular backgrounds. These findings suggest that the impact of maspin on PDAC phenotypes and molecular pathways is governed by complex regulatory mechanisms including protein–protein interactions and genetic backgrounds, as well as subcellular localization. Therefore, it is essential for future studies to establish experimental systems for more rigorous functional evaluation, combining artificial control of subcellular localization using nuclear localization signals and nuclear export signals with the generation of clones with distinct subcellular localization derived from the same parental cell line for detailed analyses.

Our findings suggested that panMaspin has a tumor-suppressive function, whereas cytMaspin promotes tumor progression in PDAC cell lines. However, these findings contradict previous reports showing that high maspin expression in PDAC promotes carcinogenesis [10,12,37,38]. In a study using resected specimens, Cao et al. reported that most PDAC cases (182/223) with diffuse positivity for maspin on immunohistochemistry exhibited cytoplasmic-only staining and had a significantly poorer prognosis than maspin-negative cases. Conversely, they found that patients with maspin staining in both the nucleus and cytoplasm had a higher proportion of well-to-moderately differentiated tumors than patients with maspin cytoplasmic staining only [10]. Similarly, Maass et al. did not provide a specific classification but reported that most PDAC cases (23/24) were maspin-positive, exhibiting diffuse strong cytoplasmic staining with nuclear staining in a subset of cells [12].

As discussed above, most research conducted to date on PDAC appears to have focused primarily on cytMaspin expression. The regulation of maspin expression and changes in its subcellular localization may be pivotal events for understanding the role of maspin in PDAC. Therefore, elucidation of the detailed mechanism of nuclear translocation of maspin is required. Although the present study highlights connections between maspin subcellular localization and distinct malignant phenotypes or signaling profiles in PDAC cell lines, it does not fully establish that maspin localization alone directly drives these biological differences. In particular, the mechanistic relationship between maspin subcellular localization and ErbB pathway activation remains unresolved and requires further investigation using more rigorously controlled experimental systems.

## 5. Conclusions

In cell lines expressing endogenous maspin, the invasive capability of the cytMaspin model was greater than that of the panMaspin model. Distinct ErbB signaling pathway activities were also observed between these cell lines, although the mechanistic relationship between maspin localization and ErbB-related signaling remains to be clarified. Furthermore, cells overexpressing panMaspin (PANC-1-masp) exhibited tumor-suppressive functions via HER2 and phosphorylated Akt suppression, whereas cells overexpressing cytMaspin (S2-020-masp) enhanced migratory capability without significantly affecting invasive activity or ErbB-related signaling. Our findings suggest that even in PDAC—where high levels of maspin expression have traditionally been considered as a poor prognostic indicator—the biological role of maspin may differ according to its subcellular localization.

## Data Availability

RNA-seq data are available in the GEO database under accession number GSE314358. The data used in this manuscript are available from the corresponding author upon email request.

## Supplementary Materials

The following supporting information can be downloaded at: https://www.mdpi.com/article/10.3390/cancers18111815/s1, Figure S1: Whole Western blot images in Figure 1. (A–D) Whole western blot images for maspin (A,B) and ACTB (C,D) in the manuscript (Figure 1A). (E–J) Whole western blot images for maspin (E,F), HSP90 (G,H), and HDAC1 (I,J) in the manuscript (Figure 1C). The molecular weight of the samples was calculated using Precision Plus Protein™ Kaleidoscope™ Prestained Protein Standards and, sizes are shown in kDa. The red dotted square indicates the proteins of interest; Figure S2: Effects of transient maspin knockdown in S2-007 cells. (A) Relative maspin mRNA expression in S2-007 cells after adenoviral transduction with scrambled control (sh-Scramble) or two independent maspin-targeting shRNAs (shMaspin-1 and shMaspin-2), as determined by quantitative real-time PCR. Expression levels were normalized to ACTB (*n* = 3). *** *p* < 0.001. Whole western blot images in Figure 2D. Whole western blot images of (B) Maspin, (C) ACTB; Figure S3: Differential gene expression and integrated pathway enrichment analyses in S2-028 (cytMaspin) and S2-007 (panMaspin) cells. (A) Volcano plot displaying differentially expressed genes in S2-028 cells (cytMaspin), representing the distribution of fold changes (*x*-axis) and significance (*y*-axis). Compared to S2-007 cells (panMaspin), the dots indicate upregulated (red), downregulated (blue), and unchanged (gray) genes. (B) TreeMap summarizing enriched gene ontology terms for upregulated genes in S2-028 cells. (C) TreeMap summarizing enriched gene ontology terms for downregulated genes in S2-028 cells. (D) KEGG pathway map highlighting differentially regulated genes between S2-028 (cytMaspin) and S2-007 (panMaspin) cells; Figure S4: Whole Western blot images in Figure 3D. Whole western blot images of (A) p-EGFR, (B) EGFR, (C) HER2, (D) p-Akt, (E) Akt, (F) ACTB; Figure S5: Whole Western blot images in Figure 3F. Whole western blot images of S2-007 cells after adenoviral transduction with scrambled control (sh-Scramble) or two independent maspin-targeting shRNAs (shMaspin-1 and shMaspin-2), (A) p-Akt, (B) Akt, (C) ACTB; Figure S6: Whole Western blot images in Figure 4. Whole western blot images for maspin (A) and ACTB (B) in the manuscript (Figure 4A). The molecular weight of the samples was calculated using Precision Plus Protein™ Kaleidoscope™ Prestained Protein Standards, and sizes (kDa) are indicated. Red dotted square indicates the proteins of interest; Figure S7: Cell proliferation and whole Western blot images of maspin-expressing established PDAC cell lines in Figure 5. (A) Cell proliferation of four cell lines transfected with ZsGreen-only (-ctrl) or maspin-ZsGreen (-masp) was measured at 6 h, 24 h, 48 h, and 72 h, and data were normalized to the corresponding 6 h value. Data are shown as the mean ± SD (*n* = 3). (B–H) Whole western blot images for established cell lines in Figure 5. (B) HER2, (C) p-Akt for PANC-1, (D) p-Akt for S2-020, (E) Akt, (F) ACTB; Table S1: List of primer and probe sets used in RT-qPCR; Table S2: List of primary antibodies used in Western blot; Table S3: List of DEGs in S2-028 compared with S2-007; Table S4: List of KEGG pathways assigned to DEGs in S2-028 compared with S2-007. Table S5: Gene sets enriched in S2-028 compared with S2-007.

## Abbreviations

The following abbreviations are used in this manuscript:

Maspin	mammary serine protease inhibitor
cytMaspin	cytoplasmic-only maspin
PDAC	pancreatic ductal adenocarcinoma
panMaspin	nuclear and cytoplasmic maspin
PanIN	pancreatic intraepithelial neoplasia
HPDE	human pancreatic duct epithelial
OS	overall survival
RPMI1640	Roswell Park Memorial Institute 1640
IMDM	Iscove’s Modified Dulbecco’s Medium
DMEM	Dulbecco’s modified Eagle’s medium
FBS	fetal bovine serum
p-EGFR	phosphorylated EGFR
p-Akt	phosphorylated Akt
PANC-1-ctrl	PANC-1-control
PANC-1-masp	PANC-1-maspin
MIA PaCa-2-ctrl	MIA PaCa-2-control
MIA PaCa-2-masp	MIA PaCa-2-maspin
PSN-1-ctrl	PSN-1-control
PSN-1-masp	PSN-1-maspin
S2-020-ctrl	S2-020-control
S2-020-masp	S2-020-maspin
PBS	phosphate-buffered saline
IF	Immunofluorescence
RT	room temperature
DEGs	differentially expressed genes
GO	gene ontology
BP	biological process
KEGG	Kyoto Encyclopedia of Genes and Genomes
DAVID	Database for Annotation, Visualization, and Integrated Discovery
REViGO	Reduce & Visualize Gene Ontology
EMT	epithelial–mesenchymal transition
