# Supplementary material for: Maspin Modulates Malignant Phenotypes Depending on Subcellular Localization in Pancreatic Ductal Adenocarcinoma Cell Lines
Source: Cancers (Basel). 2026 Jun 1;18(11):1815. doi: 10.3390/cancers18111815 (PMC13256641; doi:10.3390/cancers18111815)
Supplement: Supplementary file 1 [file cancers-18-01815-s001.zip › Supplementary Figure.pptx]

## Slide 1
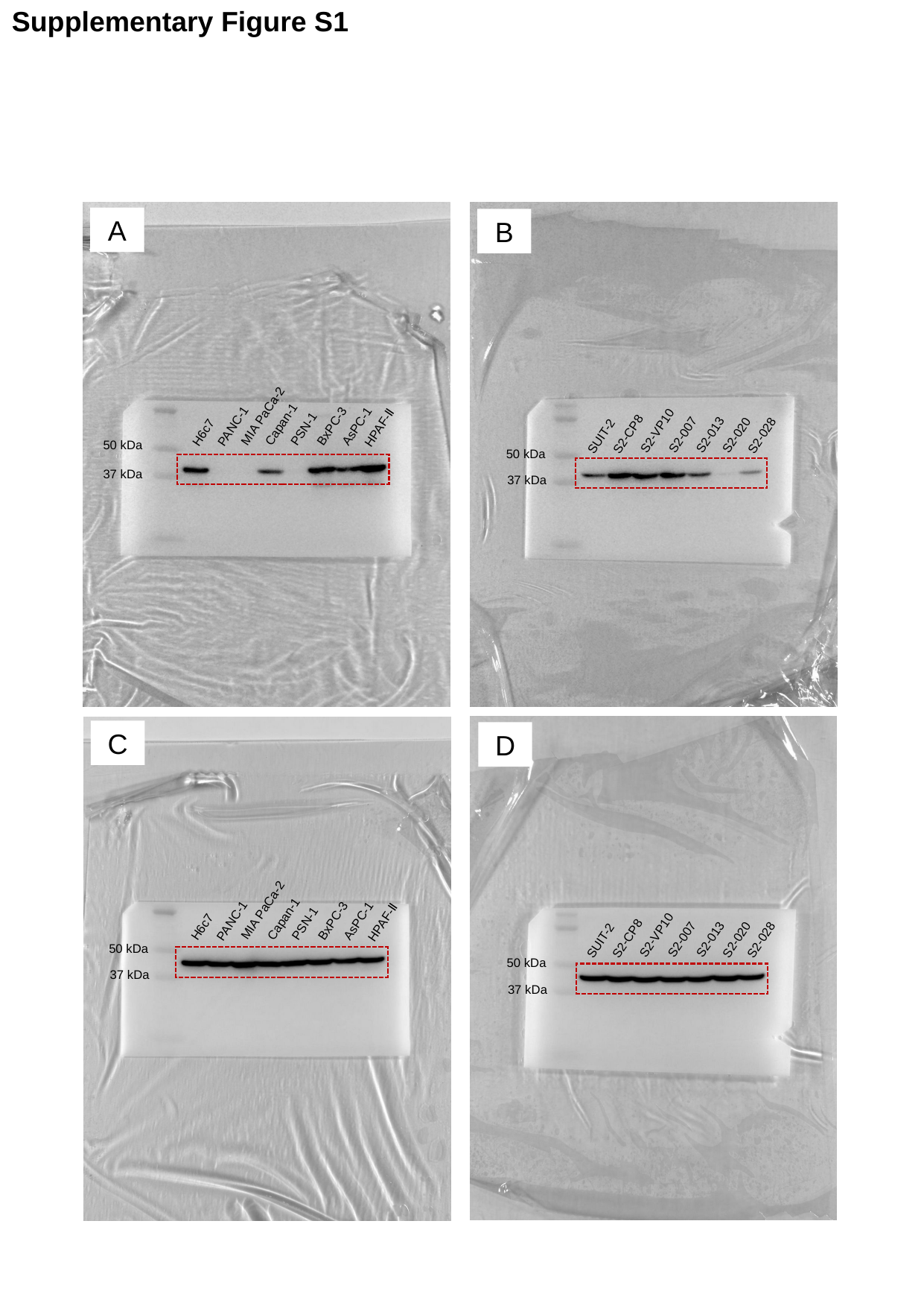

Supplementary Figure S1
A
B
MIA PaCa-2
Capan-1
PSN-1
BxPC-3
AsPC-1
HPAF-Ⅱ
H6c7
PANC-1
S2-VP10
S2-007
S2-013
S2-020
S2-028
S2-CP8
SUIT-2
50 kDa
50 kDa
37 kDa
37 kDa
C
D
MIA PaCa-2
Capan-1
PSN-1
BxPC-3
AsPC-1
HPAF-Ⅱ
H6c7
PANC-1
S2-VP10
S2-007
S2-013
S2-020
S2-028
S2-CP8
SUIT-2
50 kDa
50 kDa
37 kDa
37 kDa

## Slide 2
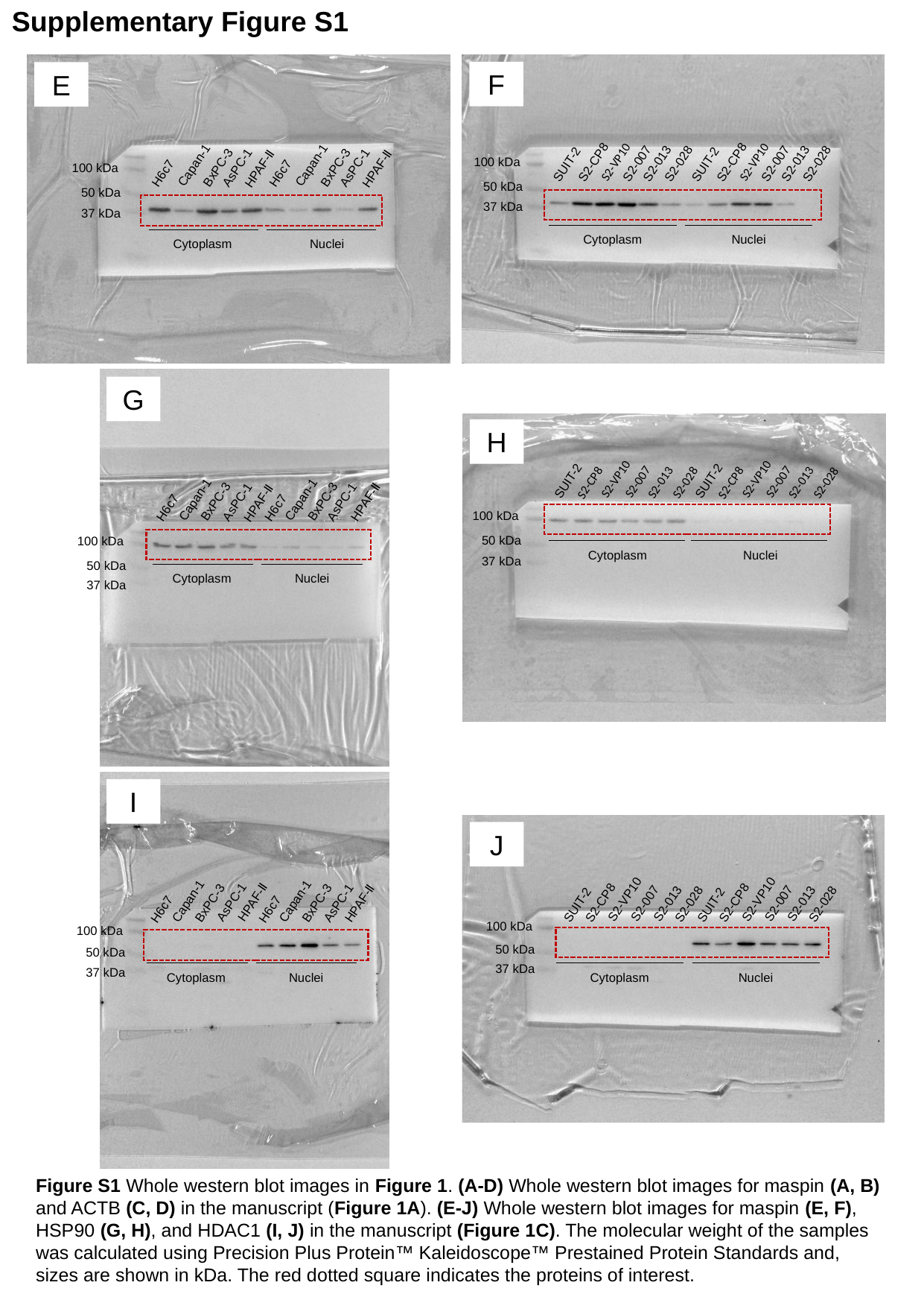

Supplementary Figure S1
F
E
S2-VP10
S2-VP10
Capan-1
Capan-1
BxPC-3
BxPC-3
AsPC-1
AsPC-1
HPAF-Ⅱ
HPAF-Ⅱ
S2-007
S2-007
S2-013
S2-013
S2-028
S2-028
S2-CP8
S2-CP8
SUIT-2
SUIT-2
H6c7
H6c7
100 kDa
100 kDa
50 kDa
50 kDa
37 kDa
37 kDa
Cytoplasm
Nuclei
Cytoplasm
Nuclei
G
H
S2-VP10
S2-VP10
S2-007
S2-007
S2-013
S2-013
S2-028
S2-028
S2-CP8
S2-CP8
SUIT-2
SUIT-2
Capan-1
Capan-1
BxPC-3
BxPC-3
HPAF-Ⅱ
AsPC-1
AsPC-1
HPAF-Ⅱ
H6c7
H6c7
100 kDa
50 kDa
100 kDa
Cytoplasm
Nuclei
37 kDa
50 kDa
Nuclei
Cytoplasm
37 kDa
I
J
S2-VP10
S2-VP10
HPAF-Ⅱ
AsPC-1
Capan-1
Capan-1
BxPC-3
BxPC-3
HPAF-Ⅱ
AsPC-1
S2-007
S2-007
S2-013
S2-013
S2-028
S2-028
S2-CP8
S2-CP8
SUIT-2
SUIT-2
H6c7
H6c7
100 kDa
100 kDa
50 kDa
50 kDa
37 kDa
37 kDa
Nuclei
Cytoplasm
Nuclei
Cytoplasm
Figure S1 Whole western blot images in Figure 1. (A-D) Whole western blot images for maspin (A, B) and ACTB (C, D) in the manuscript (Figure 1A). (E-J) Whole western blot images for maspin (E, F), HSP90 (G, H), and HDAC1 (I, J) in the manuscript (Figure 1C). The molecular weight of the samples was calculated using Precision Plus Protein™ Kaleidoscope™ Prestained Protein Standards and, sizes are shown in kDa. The red dotted square indicates the proteins of interest.

## Slide 3
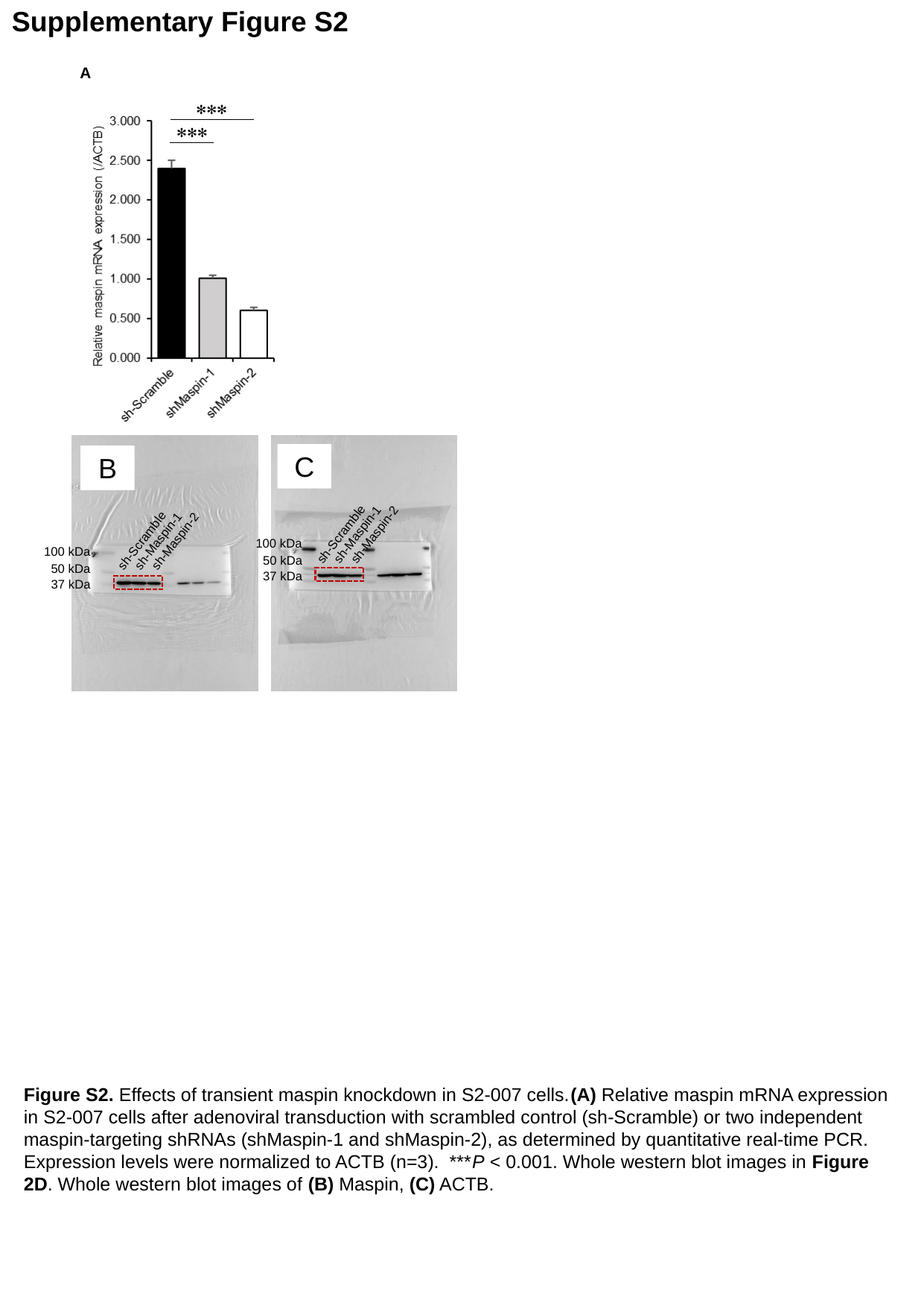

Supplementary Figure S2
A
✻✻✻
✻✻✻
C
B
sh-Scramble
sh-Maspin-1
sh-Maspin-2
sh-Scramble
sh-Maspin-1
sh-Maspin-2
100 kDa
100 kDa
50 kDa
50 kDa
37 kDa
37 kDa
Figure S2. Effects of transient maspin knockdown in S2-007 cells.(A) Relative maspin mRNA expression in S2-007 cells after adenoviral transduction with scrambled control (sh-Scramble) or two independent maspin-targeting shRNAs (shMaspin-1 and shMaspin-2), as determined by quantitative real-time PCR. Expression levels were normalized to ACTB (n=3). ***P < 0.001. Whole western blot images in Figure 2D. Whole western blot images of (B) Maspin, (C) ACTB.

## Slide 4
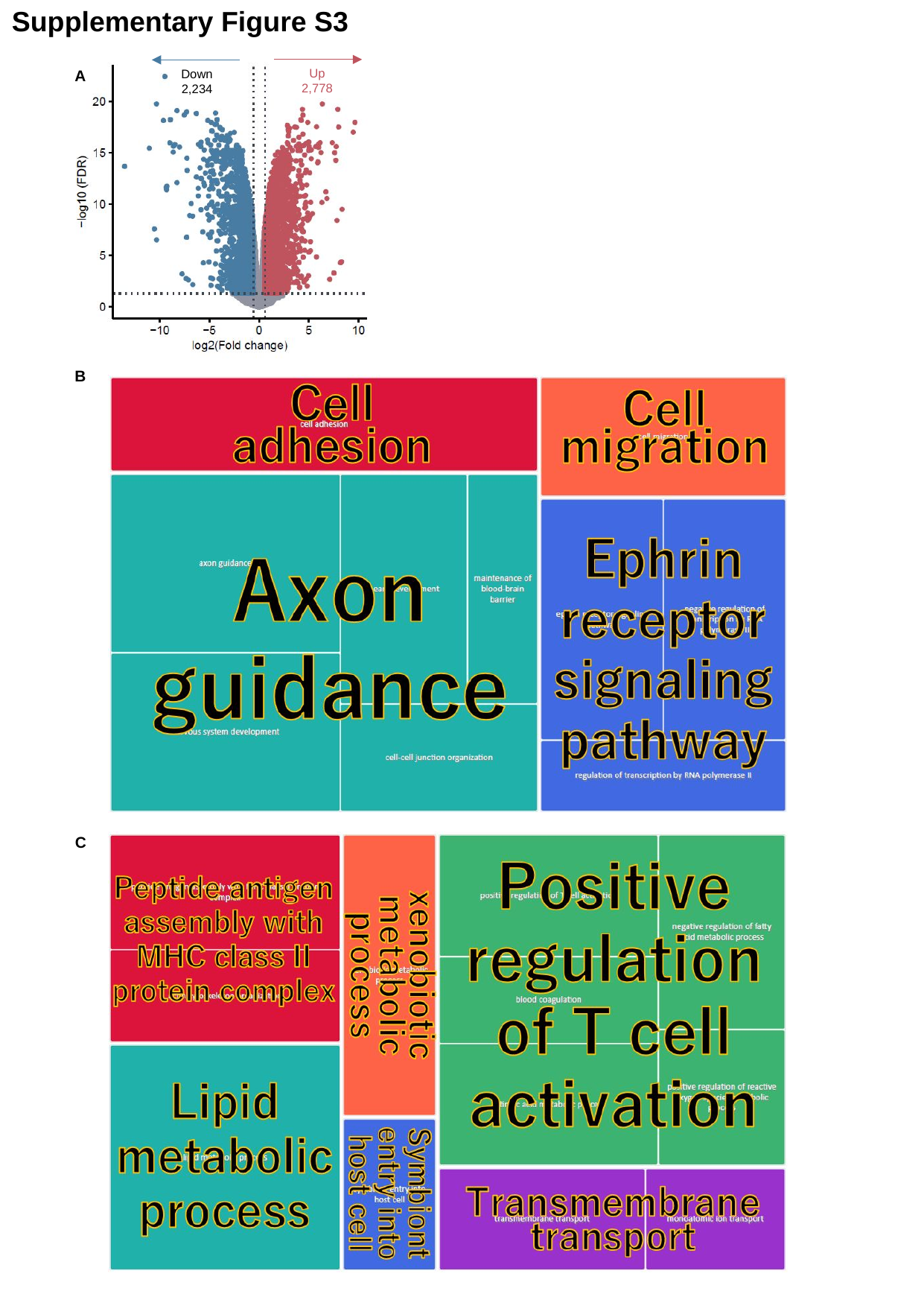

Supplementary Figure S3
Up
2,778
A
Down
2,234
B
C

## Slide 5
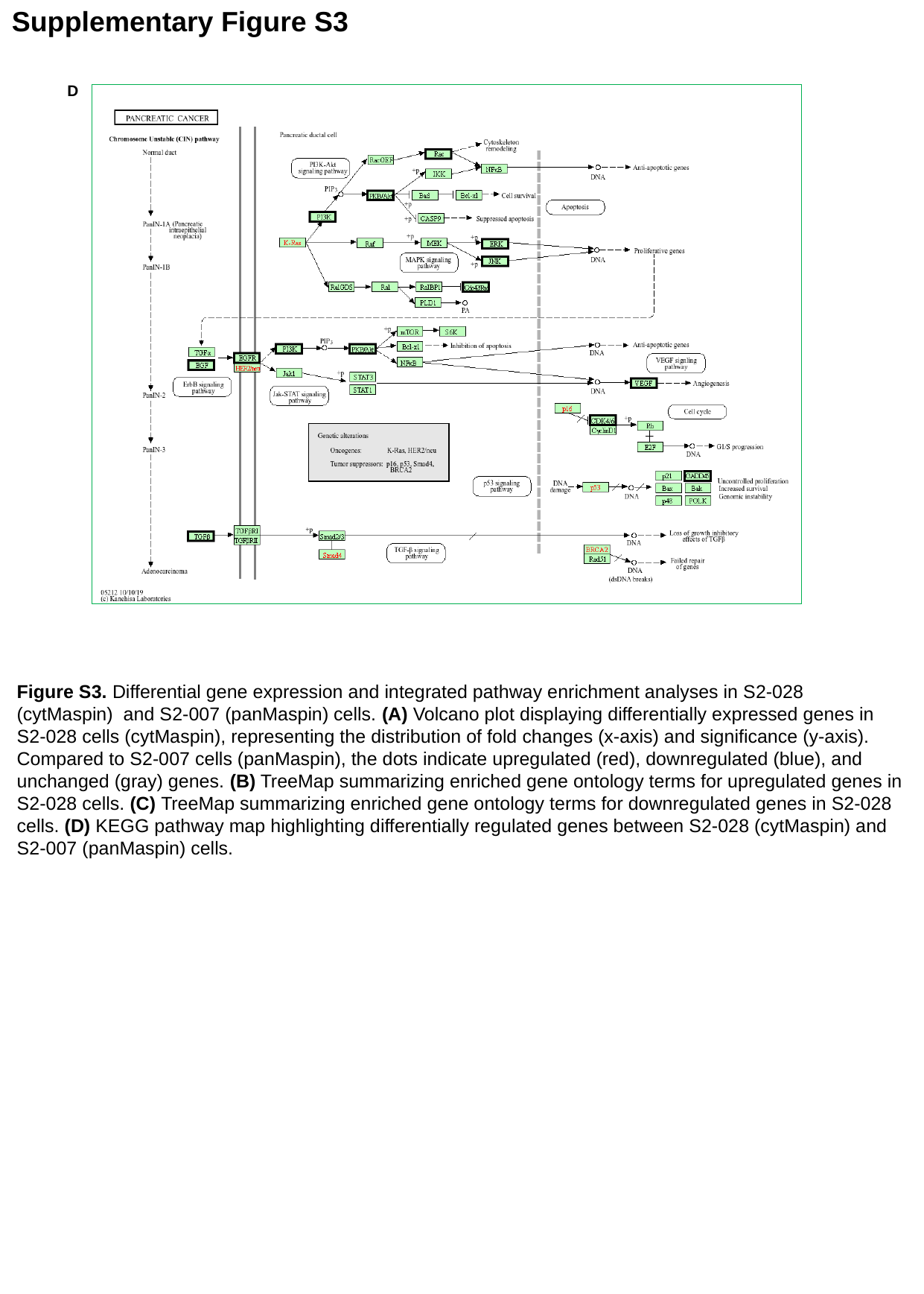

Supplementary Figure S3
D
Figure S3. Differential gene expression and integrated pathway enrichment analyses in S2-028 (cytMaspin) and S2-007 (panMaspin) cells. (A) Volcano plot displaying differentially expressed genes in S2-028 cells (cytMaspin), representing the distribution of fold changes (x-axis) and significance (y-axis). Compared to S2-007 cells (panMaspin), the dots indicate upregulated (red), downregulated (blue), and unchanged (gray) genes. (B) TreeMap summarizing enriched gene ontology terms for upregulated genes in S2-028 cells. (C) TreeMap summarizing enriched gene ontology terms for downregulated genes in S2-028 cells. (D) KEGG pathway map highlighting differentially regulated genes between S2-028 (cytMaspin) and S2-007 (panMaspin) cells.

## Slide 6
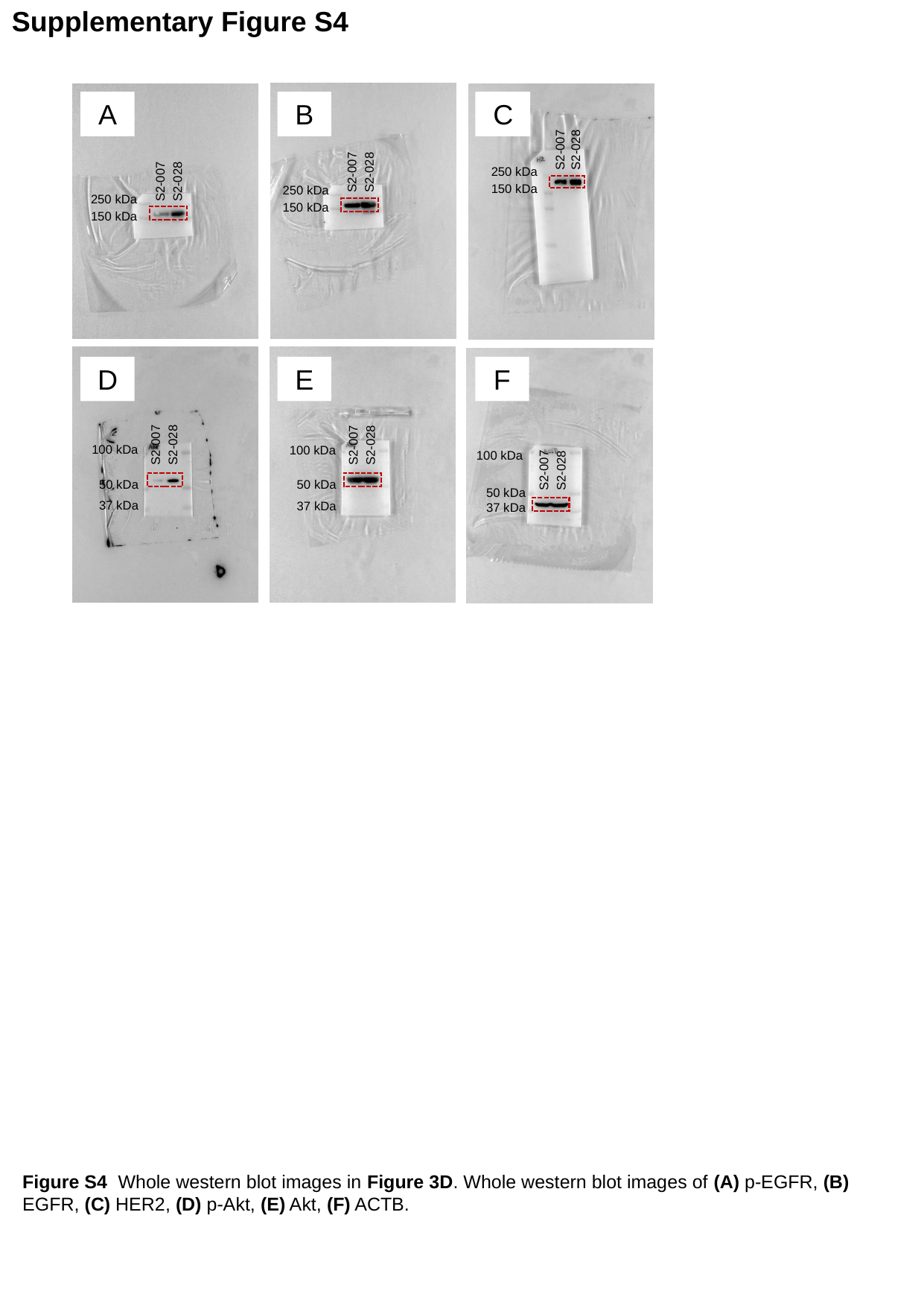

Supplementary Figure S4
B
C
A
C
S2-007
S2-028
S2-007
S2-028
250 kDa
S2-007
S2-028
150 kDa
250 kDa
250 kDa
150 kDa
150 kDa
E
D
F
S2-007
S2-028
S2-007
S2-028
100 kDa
100 kDa
100 kDa
S2-007
S2-028
50 kDa
50 kDa
50 kDa
37 kDa
37 kDa
37 kDa
Figure S4 Whole western blot images in Figure 3D. Whole western blot images of (A) p-EGFR, (B) EGFR, (C) HER2, (D) p-Akt, (E) Akt, (F) ACTB.

## Slide 7
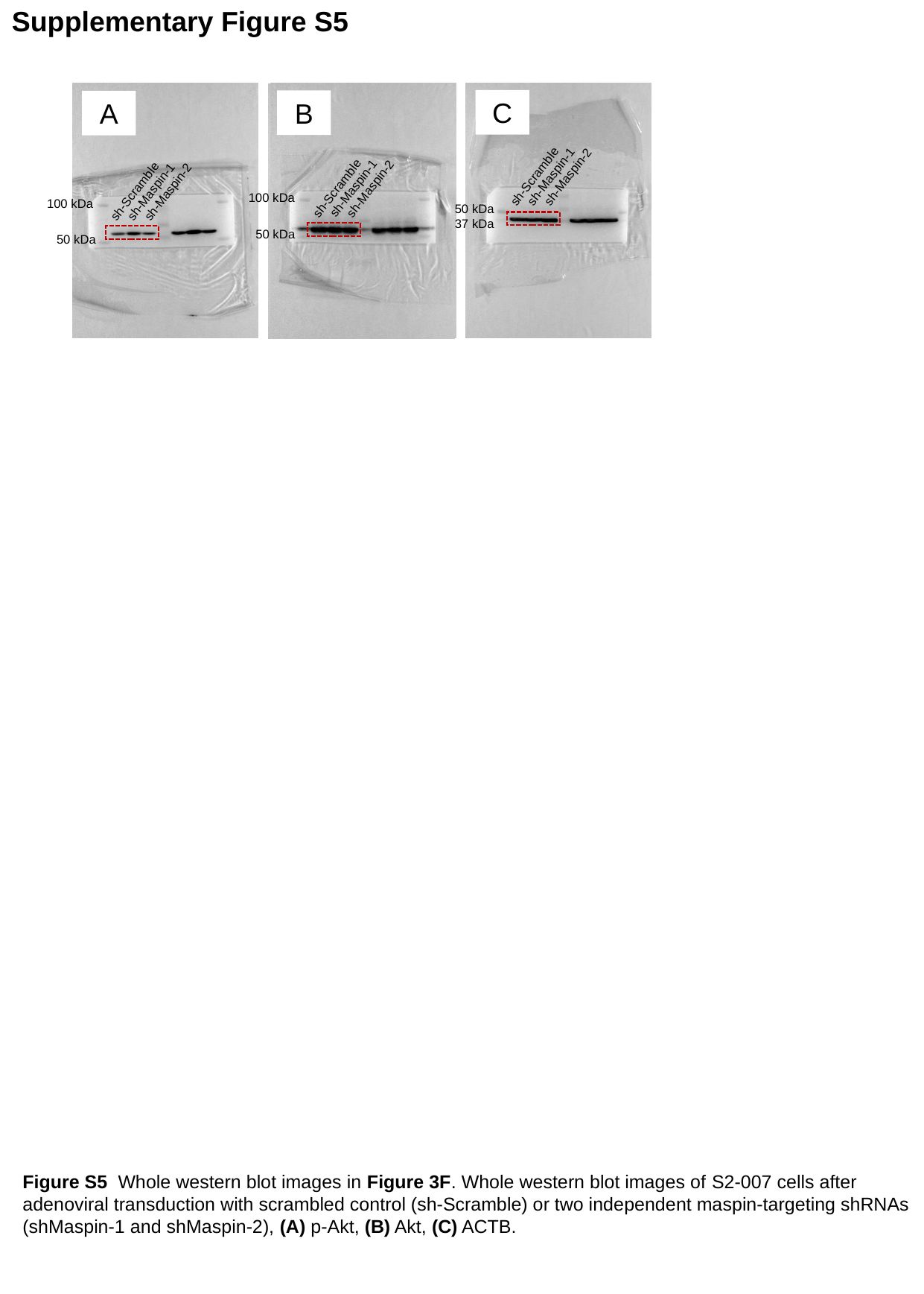

Supplementary Figure S5
C
C
C
B
B
A
F
S2-007, sh-Scramble
S2-007, sh-Maspin-1
S2-007, sh-Maspin-2
sh-Scramble
sh-Maspin-1
sh-Maspin-2
sh-Scramble
sh-Maspin-1
sh-Maspin-2
sh-Scramble
sh-Maspin-1
sh-Maspin-2
100 kDa
100 kDa
50 kDa
37 kDa
50 kDa
50 kDa
Figure S5 Whole western blot images in Figure 3F. Whole western blot images of S2-007 cells after adenoviral transduction with scrambled control (sh-Scramble) or two independent maspin-targeting shRNAs (shMaspin-1 and shMaspin-2), (A) p-Akt, (B) Akt, (C) ACTB.

## Slide 8
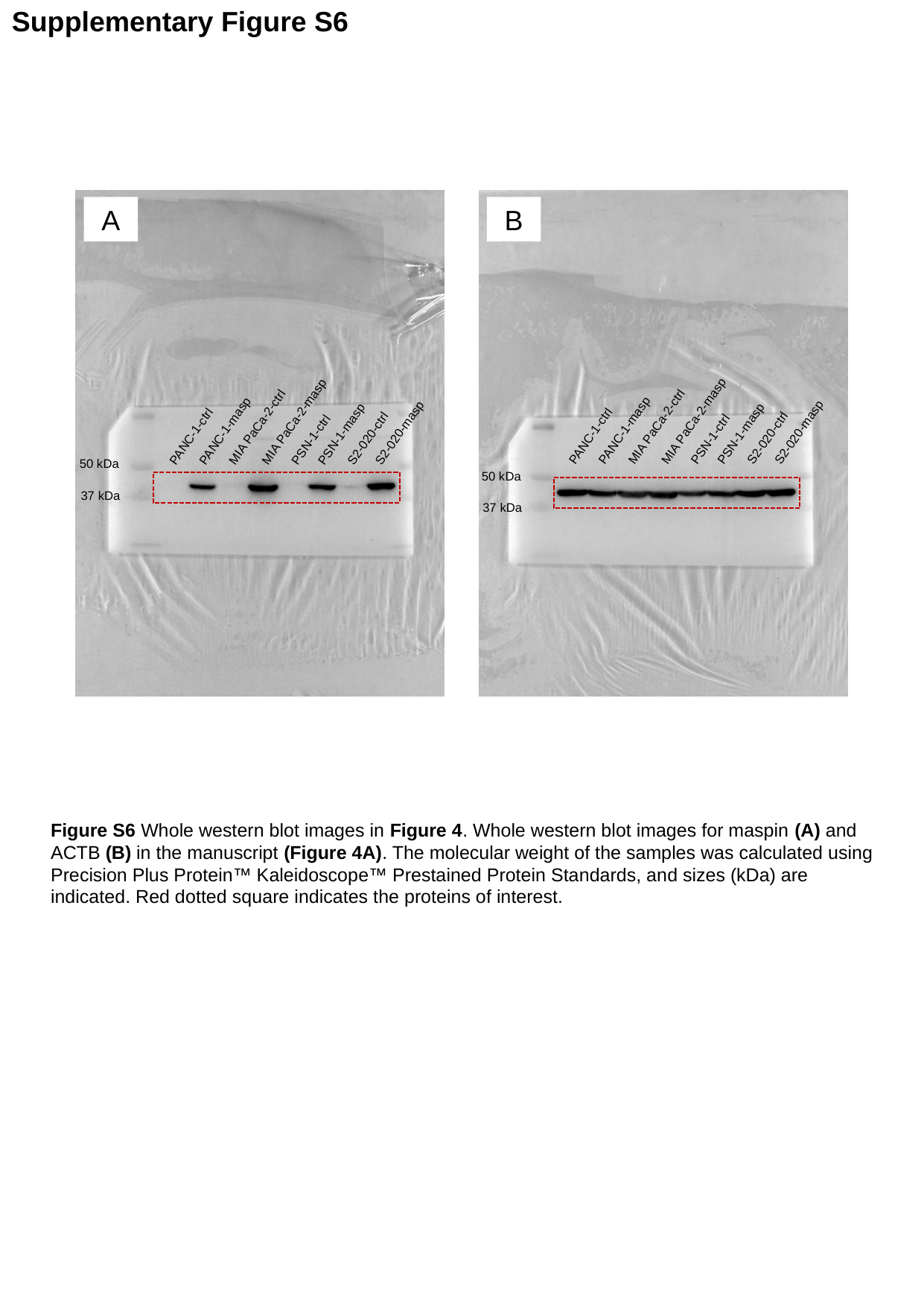

Supplementary Figure S6
A
B
PANC-1-ctrl
PANC-1-masp
MIA PaCa-2-ctrl
MIA PaCa-2-masp
PSN-1-ctrl
PSN-1-masp
S2-020-ctrl
S2-020-masp
PANC-1-ctrl
PANC-1-masp
MIA PaCa-2-ctrl
MIA PaCa-2-masp
PSN-1-ctrl
PSN-1-masp
S2-020-ctrl
S2-020-masp
50 kDa
50 kDa
37 kDa
37 kDa
Figure S6 Whole western blot images in Figure 4. Whole western blot images for maspin (A) and ACTB (B) in the manuscript (Figure 4A). The molecular weight of the samples was calculated using Precision Plus Protein™ Kaleidoscope™ Prestained Protein Standards, and sizes (kDa) are indicated. Red dotted square indicates the proteins of interest.

## Slide 9
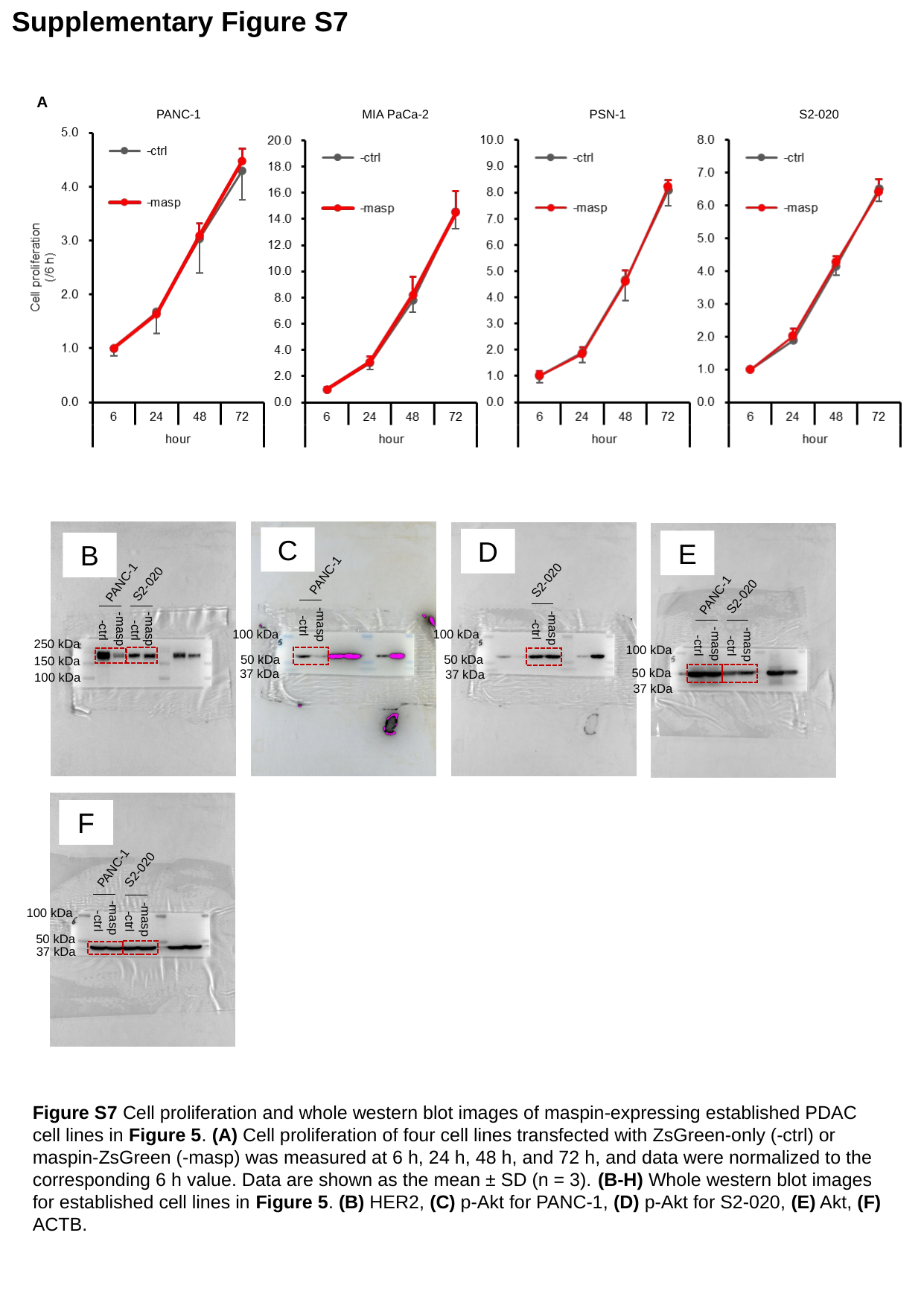

Figure 5
Supplementary Figure S7
A
PANC-1
MIA PaCa-2
PSN-1
S2-020
C
D
E
B
PANC-1
S2-020
PANC-1
S2-020
PANC-1
S2-020
-masp
-ctrl
-masp
-masp
-masp
-ctrl
-ctrl
-ctrl
100 kDa
100 kDa
250 kDa
-masp
-masp
-ctrl
-ctrl
100 kDa
50 kDa
50 kDa
150 kDa
50 kDa
37 kDa
37 kDa
100 kDa
37 kDa
F
PANC-1
S2-020
100 kDa
-masp
-masp
-ctrl
-ctrl
50 kDa
37 kDa
Figure S7 Cell proliferation and whole western blot images of maspin-expressing established PDAC cell lines in Figure 5. (A) Cell proliferation of four cell lines transfected with ZsGreen-only (-ctrl) or maspin-ZsGreen (-masp) was measured at 6 h, 24 h, 48 h, and 72 h, and data were normalized to the corresponding 6 h value. Data are shown as the mean ± SD (n = 3). (B-H) Whole western blot images for established cell lines in Figure 5. (B) HER2, (C) p-Akt for PANC-1, (D) p-Akt for S2-020, (E) Akt, (F) ACTB.
